# Brazilian guidelines on prevention of cardiovascular disease in patients with diabetes: a position statement from the Brazilian Diabetes Society (SBD), the Brazilian Cardiology Society (SBC) and the Brazilian Endocrinology and Metabolism Society (SBEM)

**DOI:** 10.1186/s13098-017-0251-z

**Published:** 2017-07-14

**Authors:** Marcello Casaccia Bertoluci, Rodrigo Oliveira Moreira, André Faludi, Maria Cristina Izar, Beatriz D. Schaan, Cynthia Melissa Valerio, Marcelo Chiara Bertolami, Ana Paula Chacra, Marcus Vinicius Bolivar Malachias, Sérgio Vencio, José Francisco Kerr Saraiva, Roberto Betti, Luiz Turatti, Francisco Antonio Helfenstein Fonseca, Henrique Tria Bianco, Marta Sulzbach, Adriana Bertolami, João Eduardo Nunes Salles, Alexandre Hohl, Fábio Trujilho, Eduardo Gomes Lima, Marcio Hiroshi Miname, Maria Teresa Zanella, Rodrigo Lamounier, João Roberto Sá, Celso Amodeo, Antonio Carlos Pires, Raul D. Santos

**Affiliations:** 1 Departamento de Medicina Interna, Faculdade de Medicina, Universidade Federal do Rio Grande do Sul (UFRGS), Rua Ramiro Barcelos, 2400, Porto Alegre, RS 90035-003 Brazil; 2 Serviço de Medicina Interna, Hospital de Clínicas de Porto Alegre (HCPA), UFRGS, Rua Ramiro Barcelos, 2350, Porto Alegre, RS 90035-903 Brazil; 3 Instituto Estadual de Diabetes e Endocrinologia Luiz Capriglione, Rua Moncorvo Filho, 90, Rio de Janeiro, RJ 20211-340 Brazil; 4 Faculdade de Medicina de Valença (FMV), Rua Sebastião Dantas Moreira, 40, Valença, RJ 27600-000 Brazil; 5 Faculdade de Medicina da Universidade Presidente Antônio Carlos (FAME/UNIPAC), Av. Juiz de Fora, 1100, Juiz De Fora, MG 36048-000 Brazil; 6 Instituto Dante Pazzanese de Cardiologia, Av. Dante Pazzanese, 500, São Paulo, SP 04012-180 Brazil; 7 Universidade Federal de São Paulo (UNIFESP), Rua Loefgren, 1350, São Paulo, SP 04040-001 Brazil; 8 UFRGS, Rua Ramiro Barcelos, 2350, Porto Alegre, RS 90035-903 Brazil; 9 Universidade de São Paulo (USP), Av. Dr. Enéas de Carvalho Aguiar, 44, São Paulo, SP 05403-000 Brazil; 10 Faculdade de Ciências Médicas de Minas Gerais, Alameda Ezequiel Dias, 275, Belo Horizonte, MG 30130-110 Brazil; 11 Universidade Federal de Goiás (UFG), 1ª Avenida, s/n, Setor Leste Universitário, Goiânia, GO 74605-020 Brazil; 12 Pontifícia Universidade Católica de Campinas (PUC-Campinas), Av. John Boyd Dunlop, s/n, Campinas, SP 13059-900 Brazil; 13 Faculdade de Ciências, Médicas da Santa Casa de São Paulo, Rua Dr. Cesário Motta Jr, 112, São Paulo, SP 01221-020 Brazil; 14 Universidade Federal de Santa Catarina (UFSC), Rua Profa. Maria Flora Pausewang, s/n, Florianópolis, SC 88040-970 Brazil; 15 Clínica de Endocrinologia e Metabologia, Av. Tancredo Neves, 1632/708, Salvador, BA 41820-020 Brazil; 16 Centro de Diabetes de Belo Horizonte, Rua Niquel, 31, Belo Horizonte, MG 30220-280 Brazil; 17 Faculdade de Medicina de São José do Rio Preto, Av. Brg. Faria Lima, 5416, São José do Rio Preto, SP 15090-000 Brazil; 18 UNIFESP, Rua Leandro Dupret, 365, São Paulo, SP 04025-011 Brazil; 19 UNIFESP, Rua Botucatu, 740, São Paulo, SP 04023-002 Brazil

**Keywords:** Diabetes mellitus, Cardiovascular prevention, Cardiovascular screening, Blood glucose, Risk factors, Coronary artery disease, Dyslipidemias, Hypertension, Antiplatelet agents

## Abstract

**Background:**

Since the first position statement on diabetes and cardiovascular prevention published in 2014 by the Brazilian Diabetes Society, the current view on primary and secondary prevention in diabetes has evolved as a result of new approaches on cardiovascular risk stratification, new cholesterol lowering drugs, and new anti-hyperglycemic drugs. Importantly, a pattern of risk heterogeneity has emerged, showing that not all diabetic patients are at high or very high risk. In fact, most younger patients who have no overt cardiovascular risk factors may be more adequately classified as being at intermediate or even low cardiovascular risk. Thus, there is a need for cardiovascular risk stratification in patients with diabetes. The present panel reviews the best current evidence and proposes a practical risk-based approach on treatment for patients with diabetes.

**Main body:**

The Brazilian Diabetes Society, the Brazilian Society of Cardiology, and the Brazilian Endocrinology and Metabolism Society gathered to form an expert panel including 28 cardiologists and endocrinologists to review the best available evidence and to draft up-to-date an evidence-based guideline with practical recommendations for risk stratification and prevention of cardiovascular disease in diabetes. The guideline includes 59 recommendations covering: (1) the impact of new anti-hyperglycemic drugs and new lipid lowering drugs on cardiovascular risk; (2) a guide to statin use, including new definitions of LDL-cholesterol and in non-HDL-cholesterol targets; (3) evaluation of silent myocardial ischemia and subclinical atherosclerosis in patients with diabetes; (4) hypertension treatment; and (5) the use of antiplatelet therapy.

**Conclusions:**

Diabetes is a heterogeneous disease. Although cardiovascular risk is increased in most patients, those without risk factors or evidence of sub-clinical atherosclerosis are at a lower risk. Optimal management must rely on an approach that will cover both cardiovascular disease prevention in individuals in the highest risk as well as protection from overtreatment in those at lower risk. Thus, cardiovascular prevention strategies should be individualized according to cardiovascular risk while intensification of treatment should focus on those at higher risk.

## Background

Since the first position statement on diabetes and cardiovascular prevention published in 2014 by the Brazilian Diabetes Society [1], important studies have been published in the area of cardiovascular assessment and prevention in patients with diabetes [2]. These studies have deeply advanced the current view on primary and secondary prevention in diabetes, and suggested new approaches on cardiovascular risk stratification, new cholesterol-lowering drugs, and new anti-hyperglycemic drugs with novel significant cardiovascular effects and mortality reduction.

To address this challenge, and in recognition of the multifaceted nature of disease, the Brazilian Diabetes Society joined the Brazilian Society of Cardiology and the Brazilian Endocrinology and Metabolism Society and gathered an expert panel formed by 28 cardiologists and endocrinologists to review the best available evidence and to draft up-to-date evidence-based guidelines with practical recommendations on both the stratification and prevention of cardiovascular disease in diabetes. The main innovations include: (1) considerations on the impact of new anti-hyperglycemic drugs and new lipid-lowering drugs on cardiovascular risk; (2) a practical risk factor-based approach to guide statin use, including new definitions of LDL-cholesterol and non-HDL-cholesterol targets; (3) an evidence-based approach to evaluate silent myocardial ischemia and subclinical atherosclerosis in patients with diabetes; (4) the best current approaches for treating hypertension; and (5) recommendation updates for the use of antiplatelet therapy. We hope these guidelines will help clinicians to improve the quality of the care provided to patients with diabetes.

## Methods

Initially, the panel members were divided into seven subcommittees to define the main topics requiring an updated position from the societies. Panel members searched PUBMED for randomized clinical trials and meta-analyses of clinical trials, and observational studies of good quality published from 1997 to 2017 using MeSH terms: [diabetes], [type 2 diabetes], [cardiovascular disease], [cardiovascular risk stratification] [coronary artery disease], [screening], [silent ischemia], [statins], [hypertension], [acetyl salicylic acid]. Low quality observational studies, meta-analyses with high heterogeneity and cross-sectional studies were not included although they might have influenced the level of evidence indicated. Expert opinion was used when the results of the search were not satisfactory for a specific item. It is important to note that it was not the aim of this position statement to include a rigorous systematic review.

A preliminary manuscript outlining recommendation grades and levels of evidence (Table 1) was then drafted. This step took several rounds of discussion among subcommittee members, who reviewed the findings and made new suggestions. The manuscript was then returned to the lead author in charge of text harmonization and inclusion of minor changes, and was subsequently submitted to further view rounds by committee members, seeking a consensus position. After this phase, the manuscript was forwarded to the editorial board for final editing and submitting for publication.

**Table 1:** Recommendation grades and levels of evidence

Grade of recommendation	
Class I	Evidence is conclusive or, if not, there is a general consensus that a procedure or a treatment is safe and efficacious
Class II	There is conflicting evidence or divergent opinion on safety, efficacy or utility of treatment or procedure
Class IIa	Opinions are in favor of the treatment or procedure. The majority of experts approves
Class IIb	Less well established efficacy, opinions are divergent
Class III	There is evidence or consensus that the treatment or procedure is not useful, efficacious or may be harmful
Levels of evidence	
A	Multiple concordant well designed randomized clinical trials or robust meta-analyses of randomized clinical trials
B	Data from less robust meta-analyses, a single randomized clinical trial or observational studies
C	Expert opinion

- These guidelines were divided into seven modules, namely:
- **Cardiovascular risk**
- Module 1: Cardiovascular risk stratification
- Module 2: Screening of subclinical atherosclerosis
- Module 3: Screening of silent myocardial ischemia
- **Cardiovascular prevention**
- Module 4: Management of hyperglycemia
- Module 5: Management of dyslipidemia
- Module 6: Management of hypertension
- Module 7: Antiplatelet therapy

## Module 1: Cardiovascular risk stratification

Patients with type 1 and 2 diabetes are divided into four broad cardiovascular risk categories—LOW, INTERMEDIATE, HIGH, AND VERY HIGH (Table 2)—based on age, presence of stratifying risk factors (SF) (Table 3), subclinical atherosclerosis (SCAT) (Table 4), or clinical atherosclerotic disease (CLAD) (Table 5). The 10-year cardiovascular event rate for low, intermediate, high, and very high risk categories were respectively: <10, 10–20, 20–30, and >30% (Table 2).

**Table 2:**
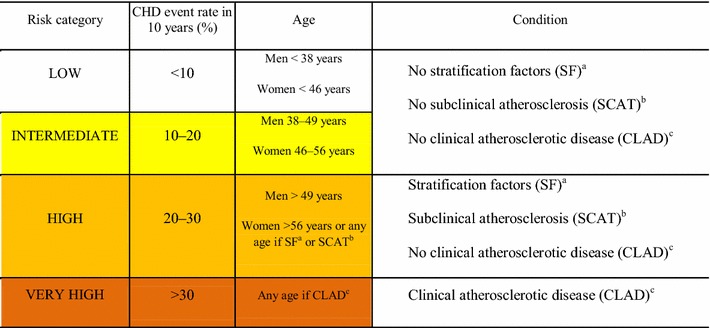
Cardiovascular risk categories in patients with diabetes

^a^Stratification factors (Table 3)

^b^Subclinical atherosclerosis (Table 4)

^c^Clinical atherosclerotic disease (Table 5)

**Table 3:** Stratifying risk factors (SF)

Age >49 years in men or >56 years in women [3]
Duration of diabetes greater than 10 years [4]^a^
Family history of premature coronary heart disease [5]^b^
Presence of IDF-defined Metabolic Syndrome [6]^c^
Treated or untreated hypertension [7]
Current smoking [8]^d^
Estimated glomerular filtration rate below 60 mL/min/1.73 m^2^ [9]
Albuminuria above 30 mg/g of creatinine [10]
Cardiac autonomic neuropathy [11]
Diabetic retinopathy [12, 13]

^a^Valid for patients in whom the onset of diabetes occurred after 18 years of age

^b^Family history of premature coronary heart disease is defined as the presence of coronary events in first-degree relatives (father, mother, or siblings) when occurring before 55 years of age in men or before 65 years of age in women

^c^The IDF definition of Metabolic Syndrome consists of: (1) abdominal circumference >90 cm for men and >80 cm for women, plus; (2) triglycerides >150 mg/dL for both men and women; (3) HDL-c < 40 mg/dL in men and <50 mg/dL in women; (4) blood pressure ≥130/85 mmHg or treatment for hypertension; and (5) a fasting blood glucose ≥100 mg/dL

^d^Current smoking is defined when the last smoking episode occurred less than 1 year before the time of stratification

**Table 4:** Subclinical atherosclerosis (SCAT)

Coronary artery calcium score (CAC) >10 U Agatston^a^
Carotid plaque (intima-media thickness >1.5 mm) [14]
Computed tomography coronary angiography (CCTA) with a definite plaque [15]^b^
Ankle-brachial index <0.9 [16]
Abdominal aortic aneurysm (AAA) [17–21]^c^

^a^When available, CAC scoring should be the preferred modality

^b^CCTA should not be performed routinely in truly asymptomatic patients

^c^Patients suffering from an AAA are at elevated risk of cardiovascular morbidity and mortality, due to common risk factors and comorbidities associated with the aneurysm

**Table 5:** Clinical atherosclerotic disease (CLAD)

Acute coronary syndrome:
Acute myocardial infarction or unstable angina
Stable angina or previous acute myocardial infarction
Atherothrombotic stroke or transient ischemic attack
Coronary, carotid, or peripheral revascularization
Peripheral vascular insufficiency or limb amputation
Severe atherosclerotic disease (stenosis >50%) in any vascular territory

The LOW and INTERMEDIATE risk categories are based solely on age and SF (Table 3). SCAT (Table 4), and CLAD (Table 5) are not present in these risk groups. As seen in a large Ontario population-based retrospective cohort study, 379,003 individuals with diabetes were included and followed up for a mean of 8 years until the occurrence of a first acute myocardial infarction or death from all causes [3]. The transition from LOW to INTERMEDIATE RISK occurred at ages 38 and 46 years respectively for men and women. The transition from INTERMEDIATE to HIGH-RISK status occurred respectively at ages: 49 and 56, for both men and women [3]. Therefore, patients with diabetes without clinical or subclinical cardiovascular disease and risk factors are considered at INTERMEDIATE RISK when aged are 38–49 years (men) or 46–56 years (women), and at LOW RISK if they are younger.

The HIGH-RISK group is defined by the presence, at any age, of at least one SF (Table 3) or one indicator of SCAT (Table 4), in the absence of CLAD (Table 5). Even in the absence of these conditions, a patient with diabetes is also considered at HIGH RISK when age is above 49 years in men or 56 years in women. Finally, the VERY HIGH-RISK group includes patients who, at any age, have CLAD as defined in Table 5.

## Module 2: Screening of subclinical atherosclerosis

### 1. Coronary artery calcification (CAC) score is associated with cardiovascular events and mortality in patients with diabetes [I, A]

#### Summary of evidence

- Coronary artery calcification (CAC) is a marker for the presence and burden of atherosclerosis, as demonstrated in anatomical studies [22]. The MESA [23] and Heinz Nixdorf [24] studies demonstrated that CAC is a predictor of coronary events and is useful for stratification of cardiovascular risk among patients in primary prevention. This is also true for patients with diabetes: the higher the CAC score, the higher the risk of cardiovascular events in subjects with diabetes [25].
- Raggi et al. [26] followed 10,377 asymptomatic individuals (903 with diabetes), who had been investigated with CAC at baseline, for a mean of 5 years. The mean CAC score was higher in patients with diabetes than in patients without diabetes (281 ± 567 vs. 119 ± 341, *p* < 0.0001). This study also showed that a higher CAC score was associated with a higher mortality rate, especially in patients with diabetes. However, the survival rate was similar to that observed in patients with and without diabetes (98.8% vs. 99.4% respectively, *p* = 0.5) when CAC was zero.
- The PREDICT study [27] followed 589 patients with diabetes without cardiovascular disease (mean age 63.1 years) for a median of 4 years. The greater the coronary calcium score, the greater the risk of cardiovascular outcomes. The area under the ROC curve (AUC-ROC) for risk determination using the UKPDS risk score was 0.63, and was increased to 0.73 when CAC was included (*p* = 0.03).

### 2. Coronary artery calcium score (CAC score) determination has the best net reclassification rate compared to other risk markers when added to clinical global risk score calculators alone. This can be especially useful to reclassify patients at INTERMEDIATE risk to higher or lower-risk categories. However, this Panel recognizes that, despite its utility, CAC score may not be easy to obtain in a large proportion of patients [IIa, B]

#### Summary of evidence

- In a large cohort study of 44,052 asymptomatic individuals referred for CAC testing, including 2384 with diabetes [28], the authors showed that cardiovascular risk was more accurately stratified with CAC in patients with diabetes. Patients in the low and moderate risk categories had a mortality rate of 39.4 deaths/1000/year when CAC was above 100. Conversely, those classified in the clinical high-risk category with no calcium present (CAC = 0) had a 10-year mortality rate of 6.59 deaths/1000/year. In the lower-risk subgroup (<5% in 10 years), 18% had CAC > 100, while in the higher risk category (>20% in 10 years), 16% had CAC = 0. In other words, CAC was able to reclassify a considerable number of low-risk patients into a high-risk category [27]. A CAC score >0 was present in 57.3% of patients in the low-risk category and in 70.6% of those in intermediate-risk categories.
- The prospective, community-based coronary artery risk development in young adults (CARDIA) study [29] recruited 5115 participants aged 18–30 years, with CAC measured at 15, 20, and 25 years after recruitment. The main outcomes were incident coronary heart disease, including fatal and nonfatal myocardial infarction, acute coronary syndrome without myocardial infarction, coronary revascularization, or CHD death. The probability of developing CAC by age 32–56 was estimated using clinical risk factors measured 7 years apart between ages 18 and 38. Participants were followed up for 12.5 years, with 57 incident CHD events and 108 incident CVD events observed. After adjusting for risk factors and treatments, those with any CAC had a fivefold increase in CHD events (hazard ratio [HR] 5.0, 95% CI 2.8–8.7) and a threefold increase in CVD events (HR 3.0, 95% CI 1.9–4.7). Within CAC score strata of 1–19, 20–99, and >100, the HRs for CHD were 2.6 (95% CI 1.0–5.7), 5.8 (95% CI 2.6–12.1), and 9.8 (95% CI 4.5–20.5), respectively. A CAC score ≥100 was associated with an incidence of 22.4 deaths per 100 participants in 12.5 years (HR 3.7, 95% CI 1.5–10.0). The presence of CAC among individuals aged 32–46 was associated with increased risk of fatal and nonfatal CHD during 12.5 years of follow-up. Thus, screening for CAC might be considered in individuals with risk factors in early adulthood to inform discussions about primary prevention.
- The MESA study was a prospective population-based cohort that investigated the prevalence and progression of subclinical cardiovascular disease in persons without cardiovascular disease at baseline, including 6814 men and women aged 45–84 years and 9.8% with diabetes, to assess the predictive accuracy and improvement in reclassification gained by the addition of CAC score (among others) over the atherosclerotic cardiovascular risk estimator (ASCVD). The authors concluded that CAC score had a modestly improved discriminative ability over ASCVD. The Harrell’s C statistic difference was significant (0.74 vs. 0.76, p = 0.04), and CAC score addition was the only marker that improved ASCVD risk score [30].

### 3. In patients with diabetes, a CAC score >10 is an indicator of increased mortality and future cardiovascular events. It is recommended that patients with diabetes with a CAC score >10 should be considered as HIGH RISK. [I, A]

#### Summary of evidence

- In a meta-analysis of eight studies including 6521 patients with diabetes [31], with a mean follow up of 5.18 years, the relative risk of the composite outcome of all-cause mortality and/or cardiovascular events with CAC > 10 vs. CAC < 10 was 5.47 (95% CI 2.59–11.53, *p* < 0.001) [31]. Nevertheless, it should be noted that significant heterogeneity was detected across studies (I^2^ = 82.4%, *p* < 0.001). CAC > 10 had a sensitivity of 94% and a specificity of 34% for the composite outcome. A higher CAC score entailed lower sensitivity and higher specificity. For example, when comparing CAC < 10 vs. CAC > 1000, sensitivity dropped to 90%, while specificity increased to 74%. For an individual with diabetes and CAC < 10, post-test probability for the composite outcome was 1.8%, representing a 6.8-fold decrease in the pretest probability of a CVD outcome. The study concluded that a CAC < 10 is helpful to detect lower-risk individuals in this population.
- The Diabetes Heart Study monitored cardiovascular mortality in 1051 patients with diabetes followed for 7.4 years. A positive association was observed between CAC and mortality in the model adjusted for age, sex, race, smoking, and LDL-C. Using the group score of 0–9 for CAC as a reference, the study found the following relative risks according to CAC severity: CAC 10–99: 1.40 (95% CI 0.57–3.74, *p* = 0.47); CAC 100–299: 2.87 (95% CI 1.17–7.77, *p* = 0.02); CAC 300–999: 3.04 (95% CI 1.32–7.90, *p* = 0.008); and CAC ≥ 1000: 6.71 (95% CI 3.09–16.87, *p* = 0.0001) [32]. Later in 2013, the same authors published an analysis of CAC score compared to traditional risk factors to predict cardiovascular mortality. CAC improved the AUC-ROC from 0.70 (95% CI 0.67–0.73) to 0.75 (95% CI 0.72–0.78). The net reclassification index (NRI) in the moderate-risk group (7–20% in 10 years) was 0.34, which means that 34% of individuals were reclassified into different risk categories [33].

### 4. CAC score outperforms carotid-artery intima-media thickness (CIMT) and ankle-brachial index (ABI) to discriminate and reclassify cardiovascular risk, at least in nondiabetic subjects. [IIa, B]

#### Summary of evidence

- The MESA study compared the performance of distinct stratification methods in an intermediate-risk population with no previous cardiovascular event (estimated Framingham risk score between 5 and 20%) [33]. In that study, calcium score (AUC for CAC plus Framingham risk score: 0.784) presented better risk discrimination compared to CIMT (AUC for CIMT plus Framingham risk score: 0.652) and ABI (AUC for ABI plus Framingham risk score: 0.650), as well as better reclassification ability (NRI for calcium score: 0.659; NRI for CIMT: 0.102; NRI for ABI: 0.036) [33]. Although patients with diabetes mellitus were not part of the study, calcium score was shown to be clearly superior to CIMT and ABI to predict risk of coronary events.

### 5. Carotid plaque can predict major adverse cardiovascular events (MACE) and reclassify risk. Adding plaque information with abnormal wall thickness (CIMT > 1.5 mm) may be useful to reclassify intermediate risk into high risk. [IIb, B]

#### Summary of evidence

- The Atherosclerosis Risk in Communities (ARIC) study followed 13,145 individuals without previous CVD (57% women, age: 54.0 ± 8.5 years, 10% with diabetes) for a mean of 15.1 years, during which 1812 CHD events occurred [34]. CIMT (categorized as <25th percentile, 25th–75th percentile or >75th percentile for sex) or plaque presence, defined in the presence of at least 2 of 3 criteria—abnormal wall thickness (CIMT > 1.5 mm), abnormal shape (protrusion into the lumen, loss of alignment with adjacent arterial wall boundary), and abnormal wall texture (brighter echoes than adjacent boundaries)—were superior for risk discrimination and reclassification in comparison with risk factors alone. According to the authors, when plaque information (abnormal wall thickness) and CIMT were considered in addition to risk factors, 8.6, 37.5, 38.3, and 21.5% of the overall sample were reclassified in the <5, 5–10, 10–20, and >20% 10-year estimated risk groups respectively. Adding plaque and CIMT reclassified 17.4, 32.8, 36.6, and 25.2% of the men and 5.1, 40.2, 38.4, and 24.9% of the women in the same risk groups.
- The prospective cohort BioImage Study enrolled 5808 asymptomatic adults without previous cardiovascular events to evaluate the role of vascular imaging in cardiovascular risk prediction [35]. All patients were evaluated for carotid plaque burden score based on a novel 3-dimensional carotid ultrasound and CAC score at baseline, and followed up for a median of 2.7 years. The main study outcome was the presence of MACE defined as cardiovascular death, myocardial infarction, and ischemic stroke. The authors analyzed the carotid plaque burden (cPB) through the sum of the areas of carotid plaques as seen along both carotid arteries and their ramifications. cPB was analyzed in tertiles. After adjustments for risk factors, and compared with individuals without any cPB, hazard ratios (HR) for MACE at tertiles 1, 2, and 3 were 0.78 (95% CI 0.31–1.91), 1.45 (95% CI 0.67–3.14), and 2.36 (95% CI 1.13–4.92) respectively. The net reclassification index (NRI) significantly improved in 23%. Thus, detection of subclinical carotid atherosclerosis improves risk prediction and reclassification compared with traditional risk factors [35].

## Module 3: Screening of silent myocardial ischemia

### 6. A resting electrocardiogram (ECG) should be considered at least annually in asymptomatic patients with diabetes at INTERMEDIATE, HIGH, and VERY HIGH RISK. [IIa, B]

#### Summary of evidence

- On the basis of expert-opinion evidence, an annual resting ECG is recommended for diabetic patients at high and very high cardiovascular risk, given its low cost, high safety, and prognostic value of ECG abnormalities, which must lead to further exploration.
- In the Epidemiology of Diabetes Interventions and Complications (EDIC) Study [36], patients with type 1 diabetes had a mean follow-up of 19 years and underwent at least one ECG annually. The presence of any major ECG abnormalities was associated with a more than twofold increased risk of CVD events (hazard ratio [HR] 2.10 [95% CI 1.26–3.48] vs. no abnormality/normal ECG, and 2.19 [95% CI 1.46–3.29] vs. no major abnormality).
- In the United Kingdom Prospective Diabetes Study (UKPDS), one in every six newly diagnosed patients with diabetes had ECG evidence of silent myocardial infarction [37].
- The MiSAD study [38] included 925 asymptomatic intermediate to high-risk patients with type 2 diabetes mellitus who underwent an ECG stress test, which, if positive, led to stress myocardial perfusion imaging (MPI). The prevalence of coronary artery disease (CAD) was 12.5% (abnormal exercise test). Of individuals with CAD, 6.4% had abnormal perfusion at MPI. Multivariate analysis showed that, in the overall population, the associated independent risk factors were age, total cholesterol, proteinuria, and, importantly, ST-T abnormalities on resting ECG, which had the highest odds ratio (9.27, CI 4.44–19.38) and was the only risk factor identified in both women and men. Abnormal MPI predicted cardiac events at 5 years (HR 5.5, 95% CI 2.4–12.3, *p* < 0.001). The relevance of ST-T abnormalities on resting ECG as a predictor of silent CAD highlights the importance of performing periodic resting ECGs in patients with type 2 diabetes.

### 7. Universal screening for coronary artery disease with stress induction of myocardial ischemia does not improve outcomes and is NOT RECOMMENDED in truly asymptomatic diabetic patients when in the absence of resting ECG abnormalities, even in the presence of a high-risk condition for cardiovascular events. [III, A]

#### Summary of evidence

- The detection of ischemia in asymptomatic diabetics (DIAD) multicenter randomized trial evaluated whether detection of silent myocardial ischemia in asymptomatic patients with diabetes could reduce cardiovascular events. The participants were randomized to undergo routine screening for detection of silent ischemia using adenosine stress myocardial perfusion single-photon emission computed tomography (SPECT) or no screening. A total of 1123 asymptomatic diabetic patients were randomized. After a mean follow-up of 4.8 years, a non-significant reduction in the overall cardiac event rate was detected in the screened vs. unscreened group, with HR of 0.88 (95% CI 0.44–1.88) [39].
- A prospective, multicenter randomized trial—do you need to assess myocardial ischemia in type-2 diabetes (DYNAMIT) study [40]—evaluated screening for silent myocardial ischemia using a bicycle exercise test or dipyridamole stress SPECT in 631 asymptomatic diabetic patients with no evidence of coronary artery disease. The study was discontinued prematurely because of difficulties in recruitment and a lower-than-expected event rate. There were no significant differences between the screening and usual-care groups for the main outcome (HR 1.00, 95% CI 0.59–1.71). A meta-analysis of the DYNAMIT and DIAD trials [39] produced similar results, with narrower confidence intervals for each endpoint.
- The BARDOT trial [41] was a prospective multicenter study evaluating the prevalence, progression, treatment, and outcome of silent coronary artery disease (CAD) in 400 asymptomatic patients with diabetes at high coronary risk, without history or symptoms of CAD. Patients underwent myocardial perfusion SPECT (MPS) at baseline and after 2 years [41]. Patients with normal MPS received usual care, while those with abnormal MPS received medical or combined invasive and medical management. An abnormal MPS was found in 22% of patients. Normal-MPS patients had a low rate of first manifestations of CAD compared with patients with abnormal MPS at baseline. Patients with normal MPS had 2-year rates of MACE, cardiac death, and of new ischemia or new scar of 2.9, 0.7, and 3.2% respectively. Patients with abnormal MPS had a sevenfold higher rate of progression to “overt CAD,” independent of therapy [41]. However, although the BARDOT trial results suggested screening and treating high-risk patients on the basis of MPS, it is important to note that only about 20% of patients with an abnormal MPS would be advised to receive anti-ischemic therapy. The findings of the Bardot study are preliminary and still require confirmation. A combined medical and invasive strategy may reduce scintigraphic but not symptomatic CAD progression compared with medical therapy alone. Thus, universal screening cannot be currently advised in high-risk patients until more robust data are available.

### 8. Consider investigation for myocardial ischemia in asymptomatic patients with diabetes when resting ECG abnormalities are present and in patients who exhibit typical or atypical cardiac symptoms (unexplained dyspnea, atypical chest pain or discomfort), evidence of associated vascular disease (carotid bruits, transient ischemic attack, stroke, peripheral arterial disease) and a very high CAC score (>400), when available. [IIa, B]

#### Summary of evidence

- In a sub-study of the 30-year UKPDS [37], data from 5102 diabetic patients were analyzed through Cox proportional hazards regression to examine outcomes by silent myocardial ischemia (SMI) status. Of 1967 patients with complete baseline data, 326 (16.6%) had ECG evidence of SMI at enrollment. Around one in six UKPDS patients with newly-diagnosed T2D had evidence of SMI, which was independently associated with an increased risk of fatal MI and all-cause mortality.
- Raggi et al. [26] conducted a 5-year follow-up of 10,377 asymptomatic individuals (903 with diabetes) with a baseline CAC score available. The authors used Cox proportional hazard models, with and without adjustment for other risk factors, to predict all-cause mortality as the primary endpoint. All-cause mortality was increased in asymptomatic patients with diabetes in proportion to the screening CAC. In a risk-adjusted model, there was significant interaction of CAC with diabetes (*p* < 0.00001), indicating that for every increase in CAC, there was a greater increase in mortality for diabetic compared to nondiabetic subjects. The mortality of diabetic patients with CAC > 400 in the study was around 10% in 4–5 years, greater than that of nondiabetics.

### 9. Exercise ECG should be considered as the initial test for investigation of ischemia in most symptomatic patients. Exceptions are when resting ECG abnormalities preclude interpretation of exercise stress testing and in patients who are unable to exercise. In those cases, pharmacological stress echocardiography, myocardial perfusion imaging (MPI), coronary computed tomography angiography (CCTA), and stress perfusion cardiac magnetic resonance imaging are reasonable options. [IIa, C]

#### Summary of evidence

- The treadmill stress test is widely used for CAD detection in the general population because it is easily performed, has relatively good predictive value, and is inexpensive. In diabetic patients, the negative predictive value of the stress ECG is 87%, with 75% specificity. Lyerly et al. [42] studied 2854 men with documented diabetes mellitus (mean age 49.5 years) who completed a maximal treadmill exercise test with a mean follow-up of 16 years. Those with normal ECG presented the highest CHD-free survival. Those with abnormal ECG and those who were unable to perform maximal exercise had lower CHD-free survival rates. Stress SPECT with thallium or MIBI provides a wide range of information, including ischemia location and extension and left ventricular function, helping physicians appreciate the severity of CAD. This modality can be coupled with pharmacologic agents (dipyridamole, adenosine) for stress induction. In individuals with diabetes, SPECT has higher sensitivity (80–90%) and specificity (75–90%) than the ECG stress test [43]. Another alternative for SMI screening is stress echocardiography using exercise or drugs such as dobutamine. Stress echocardiography detects wall motion abnormalities during stress and provides information on ischemia intensity and left ventricular function. Sensitivity and specificity are 81 and 85% respectively in asymptomatic diabetic patients [44]. CMRI perfusion imaging, with sensitivity of 86.5% and specificity of 83.4% to detect angiographically significant coronary stenosis (>50% left main coronary artery or >70% branch disease), is an alternative for patients who cannot exercise [45].

### 10. Coronary computed tomography angiography (CCTA) should NOT be used routinely in ASYMPTOMATIC patients with diabetes, since it does not seem to reduce cardiovascular event risk when used for risk stratification of this population. [III, B]

#### Summary of evidence

- The FACTOR 64 study [46] evaluated whether CCTA was beneficial to reduce clinical outcomes in asymptomatic patients with type 1 or 2 diabetes. Patients with diabetes were included if disease duration was at least 5 years. The patients were randomly assigned to CCTA or optimal diabetes care, and the result of CCTA was used for clinical decision-making. Non-screening patients received standard-of-care treatment for existing risk factors, and physicians were encouraged to reach therapeutic goals in accordance with current guidelines (glycated hemoglobin <7.0%, LDL-c < 100 mg/dL, systolic blood pressure < 130 mmHg). Patients in the screening CCTA arm with normal coronary arteries remained on standard-of-care therapy. Patients who exhibited mild or severe proximal lesions or distal lesions or a CAC score >10 were advised to pursue more aggressive treatment targets (LDL-c < 70 mg/dL, HDL-c > 50 mg/dL, triglycerides <150 mg/dL, glycated hemoglobin <6.0%, and systolic blood pressure <120 mmHg). Patients with severe stenosis underwent invasive coronary angiography, and the decision regarding revascularization was based on the judgment of the assistant physician. Patients with moderate lesions underwent evaluation of myocardial ischemia. Nine hundred patients were randomized, 452 to CCTA, with a mean follow-up of 4 years. Mean duration of diabetes in the group not undergoing CCTA was 13.5 years, vs. 12.3 years in the CCTA arm. The primary endpoint rate (total mortality, nonfatal MI, or unstable angina) was similar, with 28 events (6.2%) in the CCTA group vs. 34 events (7.6%) in the control group (HR 0.80, 95% CI 0.49–1.32, *p* = 0.38). No differences were observed for the secondary endpoint (major ischemic cardiovascular events). In fact, the observed event rate was lower than expected for the sample size, which may explain the negative results. Patients with diabetes in whom risk factors were well controlled did not benefit from CCTA screening as a preventive measure to reduce cardiovascular event risk. Thus, CCTA cannot be recommended for screening of asymptomatic patients with diabetes at this time.

### 11. In patients at LOW or INTERMEDIATE risk categories, with atypical symptoms, coronary computed tomography angiography (CCTA) may be considered to rule out myocardial ischemia, as it has a good negative predictive value. [IIb, B]

#### Summary of evidence

- Hadamitzky et al. [47] evaluated the role of CCTA for prediction of cardiovascular events in 140 subjects with diabetes and 1782 without diabetes followed for a mean of 33 months. Participants presented with atypical symptoms of CHD or other risk factors. Those with diabetes and a high plaque burden, as characterized by high number of coronary segments with atherosclerotic plaque (calcified or not), had a much higher event rate than those without diabetes (1.8% vs. 0.5% per year). Plaque burden was the best marker of coronary events, even when adjusted for calcium score.

## Module 4: Management of hyperglycemia

*Targets*

### 12. In non-pregnant adult patients with type 1 or 2 diabetes mellitus, and in the absence of severe cognitive impairment or reduced life expectancy, the recommended target for glycemic control is a HbA1c below 7.0%. [I, A]

#### Summary of evidence

- The diabetes control and complications trial (DCCT) [48] and the United Kingdom Prospective Diabetes Study (UKPDS) [49] demonstrated that achieving an HbA1c below 7% reduces microvascular complications in type 1 and type 2 diabetes. In subjects with type 1 diabetes, implementing intensive glycemic control targeting an HbA1c below 7% in the first 6 years of diabetes can promote a 57% reduction in nonfatal myocardial infarction, stroke, and death from cardiovascular disease on long-term follow-up (9 years), as seen in the DCCT/EDIC study [50, 51]. Similarly, in type 2 diabetes, intensive glycemic control decreases cardiovascular outcomes in the long term (after 10 years) when implemented in recently diagnosed patients [52].
- Lower HbA1c targets were evaluated in three randomized clinical trials: action to control cardiovascular risk in diabetes (ACCORD) [53], action in diabetes and vascular disease: preterax and diamicron modified release controlled evaluation (ADVANCE) [54], and the veterans affairs diabetes trial (VADT) [55]). These trials did not detect reduction in cardiovascular outcomes when intensive control (HbA1c < 6.5%) was implemented. The ADVANCE study (*n* = 11,140), ACCORD (*n* = 10,251), and VADT (*n* = 1791) evaluated patients with type 2 diabetes and previous cardiovascular disease or risk factors and diabetes (mean duration 8–11.5 years), assessing incidence of cardiovascular disease after intensive vs. conventional treatment. The final mean HbA1c was 6.5 vs. 7.3% (ADVANCE), 6.4 vs. 7.5% (ACCORD), and 6.9 vs. 8.4% (VADT). In the ACCORD trial, but not in the other studies, a 22% increase in all-cause mortality followed intensive treatment.

### 13. Less stringent HbA1c targets (below 8.0%) are reasonable in patients with known history of severe and frequent hypoglycemic events, long-standing disease, short life expectancy, major comorbidities, and established vascular complications, as well as in less motivated, non-adherent patients and in those with diminished self-care capacity, limited resources, and a limited support system. [IIa, B]

#### Summary of evidence

- Tight glucose control may be harmful in many patients, particularly the elderly and those with other illnesses, especially cardiovascular diseases [56]. Intensive glycemic control does not lead to improved microvascular outcomes for at least 8 years. Data from randomized controlled trials suggest that intensive glycemic control immediately increases the risk of severe hypoglycemia 1.5- to 3-fold [57].
- Observational data from emergency admissions showed a consistent increase in severe hypoglycemia over one decade, especially in type 2 diabetes patients with lower HbA1c, more comedication, and more concomitant diseases [58, 59]. Hypoglycemia in these patients has been associated with increased mortality, higher risk of dementia, falls, fall-related fractures, cardiovascular events, and poor quality of life [60]. Mechanisms by which acute hypoglycemia may trigger ischemia, arrhythmia, and cardiovascular events include increases in epinephrine and norepinephrine levels, which may induce increased cardiac rate and/or contractility, thus heightening myocardial oxygen consumption, while also precipitating vasoconstriction and platelet aggregation. Moreover, acute hypoglycemia in the presence of hypokalemia prolongs cardiac repolarization and increases the QT interval, favoring a proarrhythmic state [60].
- In patients with diabetes from a Brazilian multicenter registry followed for 12 months, failure to reach HbA1c targets was associated with poorer event-free survival (all-cause mortality, nonfatal cardiac arrest, myocardial infarction, or stroke) as compared to good metabolic control (*p* < 0.041). In that study, HbA1c targets of 8.0 and 7.0% were considered in patients without a previous cardiovascular event vs. those with a previous cardiovascular event [61].
- Patients with limited resources and a limited support system, those with lower motivation, non-adherent patients, and those with diminished self-care capacity are not candidates for strict glucose control, as the risk of hypoglycemia tends to be higher [62].
- Considering the high risk of hypoglycemia with strict metabolic control, especially in elderly patients and in those in which this adverse effect may be more harmful, individualized targets should be pursued in patients with a known history of severe and frequent hypoglycemic events, longstanding disease, short life expectancy, major comorbidities, and established vascular complications [63]. Considering these data and the results of observational studies, the harms associated with an HbA1c target lower than 7.5% or higher than 9% are likely to outweigh the benefits in most adults older than 65 years [57, 64].
- Data to guide this type of individualized treatment are derived from weak evidence. However, the high frequency of risk factors for hypoglycemia and its adverse impact, as well as the marginal benefits of tight control in individuals with short life expectancy, suggest a need to reduce overtreatment, particularly among the elderly and the other groups cited above [56, 60, 64].

*Hospitalized patients*

### 14. In hospitalized patients with acute myocardial infarction, it is recommended that blood glucose be maintained in the 130–200 mg/dL range by continuous intravenous insulin infusion, followed by good long-term metabolic control. [I, B]

#### Summary of evidence

- The DIGAMI [65] study included 620 patients with diabetes and acute myocardial infarction (AMI) and used the following strategies: IV infusion of insulin and glucose in the first 24 h with a glycemic target of 126–196 mg/dL, subcutaneous administration of insulin four times daily for 3 months, vs. standard insulin therapy as clinically indicated at the time of the study. Treatment with insulin in the acute phase produced better glycemic control during hospitalization, at 3 months and at 1 year, as well as lower mortality rates at 1 and 3.4 years of follow-up.
- In the DIGAMI-2 trial [66], use of insulin during hospitalization and after discharge was compared to insulin therapy only during hospitalization and usual treatment throughout the period. Glycemic control and cardiovascular outcomes were similar in the two groups.
- In the HI-5 study [67], 240 patients with diabetes and glucose ≥140 mg/dL were included at hospital admission for AMI and randomized to strict glycemic control (target glycemia 72–180 mg/dL) with insulin plus intravenous glucose infusion for at least 24 h or conventional therapy. After discharge, the patients were managed by their physician, with a recommendation to maintain HbA1c < 7%. The groups had similar in-hospital mortality rates.

### 15. In patients undergoing cardiac surgery, it is recommended that blood glucose be maintained in the 120–150 mg/dL range through continuous intravenous insulin infusion during the hospitalization period. [I, A]

#### Summary of evidence

- Hyperglycemia before or after cardiac surgery has been associated with increased risk of complications (death, prolonged mechanical ventilation, renal failure, stroke, and deep sternal infection) [68, 69].
- The observational Portland Diabetes Project study evaluated the relationship between hyperglycemia and adverse outcomes of cardiac surgery in patients with diabetes. In the study, continuous intravenous insulin, adjusted by frequent blood glucose tests was used based on a standardized protocol conducted by nurses. Initial glucose target was 150–200 mg/dL. This was later changed to 125–175 mg/dL and then to 100–150 mg/dL because other studies were identifying the need to normalize blood glucose reduction outcomes. The use of this protocol vs. subcutaneous insulin according to glucose levels (historical control) was associated with reduced rates of infection [70] and death in about 50% of patients [71].
- A randomized controlled trial with surgical intensive coronary unit patients (63% cardiac surgery and 13% diabetes) showed benefit of intensive glycemic control (insulin infusion glycemic target 80–110 mg/dL) vs. usual glycemic control (180–200 mg/dL) in mortality, infection, acute renal failure requiring hemodialysis, blood transfusion, and polyneuropathy in critically ill patients. However, intensive glycemic control was associated with higher rates of hypoglycemia [72].
- Nevertheless, the multicenter NICE SUGAR Study, conducted in medical (63%) and surgical intensive coronary units (37% of patients respectively; 20% with a history of diabetes), showed that intensive glycemic control (target < 108 mg/dL) vs. usual control (140–180 mg/dL) increased mortality and hypoglycemia rates [73]. A meta-analysis including data from the NICE SUGAR study, with separate analysis of clinical and surgical ICUs, showed that tight glucose control did not reduce mortality in the clinical ICU, but may bring benefit to surgical patients when target blood glucose is <150 mg/dL [74]. A small RCT comparing two glycemic targets (90–120 mg/dL vs. 120–180 mg/dL) in patients with diabetes undergoing coronary artery bypass grafting showed increased risk of hypoglycemia and absence of benefit with more strict blood glucose control [75].

### 16. A basal plus bolus correction insulin regimen (a strategy using multiple doses of long- and short-acting insulins) is a reasonable option for correcting hyperglycemia in hospitalized, non-critically ill diabetic patients. [IIa, B] The use of sliding-scale insulin in the inpatient hospital setting is discouraged. [III, C]

#### Summary of evidence

- Hyperglycemia in in-hospital patients with diabetes is very common. Retrospective and randomized controlled trials in surgical populations have reported that hyperglycemia of diabetes is associated with increased length of stay, hospital complications, resource utilization, and mortality [76, 77].
- A randomized controlled trial showed that basal-bolus treatment (glargine and glulisine) improved glycemic control and reduced hospital complications (wound infection, pneumonia, acute renal failure, and bacteremia) compared with sliding-scale insulin (glulisine) in general surgery patients with type 2 diabetes [78].
- Some RCTs were performed in type 2 diabetic patients hospitalized for nonsurgical conditions. In this population, basal–bolus treatment (glargine and glulisine or NPH and regular) also improved glycemic control compared with sliding-scale insulin [79, 80].

*Outpatient treatment: monotherapy*

### 17. In patients with recently diagnosed type 2 diabetes, metformin plus non-pharmacological therapy including physical activity and targeted nutrition therapy for weight control is recommended as first-line therapy. [I, A]

#### Summary of evidence

- Metformin has a favorable efficacy and safety profile, with important metabolic effects and cardiovascular benefits. Due to its effect in reducing cardiovascular events and mortality, its efficacy in blood glucose reduction with low incidence of hypoglycemia, low cost, tolerable adverse effects, and no association with weight gain, it is the current first-line agent of choice for treatment of hyperglycemia in type 2 diabetes [81]. Titration or addition of further hypoglycemic drugs should be implemented as soon as possible to avoid inertia in achieving glucose targets.

### 18. In patients who do not tolerate metformin, any other antidiabetic drug can be recommended as monotherapy, except if contraindicated. [I, C]

#### Summary of evidence

- The UKPDS analyzed 5102 recently diagnosed type 2 diabetes patients followed up from 1977 to 1997 and found that intensive glycemic control with sulfonylurea or insulin therapy decreases progression of microvascular disease and may also reduce the risk of heart attacks. In obese patients, the UKPDS showed that metformin has similar efficacy to sulfonylureas for glucose control [52, 82, 83].
- UKPDS 34 investigated whether intensive glucose control with metformin has any specific advantage or disadvantage. A subgroup analysis compared 411 recently diagnosed overweight (>120% ideal bodyweight) type 2 diabetes patients treated with diet alone versus 342 patients using metformin, aiming for a fasting plasma glucose <110 mg/dL, and found a relative risk reduction (RRR) of 32% (*p* = 0.002) of any diabetes-related complications, a 42% RRR for any death related to diabetes (*p* = 0.017), and a 36% RRR for all-cause mortality (*p* = 0.011).

### 19. In patients with renal impairment, possible substitutions of anti-hyperglycemic drugs for type 2 diabetes are indicated in Table 6

*Outpatient treatment: second agent*

**Table 6:** Renal function adjustments of anti-hyperglycemic drugs

Drug	Maximal daily dose	Estimated GFR (mL/min)		
		45–60	30–45	<30
Insulin	Variable	NN	NN	NN
Pioglitazone	45 mg	NN	NN	NN
Linagliptin	5 mg	NN	NN	NN
Sitagliptin (mg)	100	50	50	25
Vildagliptin	50 mg bid	50 mg	50 mg	50 mg
Saxagliptin (mg)	5	2.5	2.5	2.5
Alogliptin (mg)	25	12.5	12.5	6.25
Metformin	<2550 mg	<2000 mg/day	<1000 mg/day	NR
Glimepiride	8 mg	1 mg	1 mg	NR
Gliclazide	120 mg	NN	NN	NR
Glibenclamide	20 mg	NR	NR	NR
Nateglinide	120 mg/meal	60 mg/meal	60 mg/meal	NR
Repaglinide (mg/meal)	1	0.5	0.5	0.5
Acarbose	300 mg	150 mg	150 mg	NR
Exenatide	10 mcg bid	NN	5 mcg bid	NR
Liraglutide	1.8 mg	NN	NN	NR
Lixisenatide	20 mcg	NN	NR	NR
Dulaglutide	1.5 mg/week	NN	NN	NR
Canagliflozin	300 mg	100 mg	NR	NR
Empagliflozin	25 mg	NN	NN	NR
Dapagliflozin	10 mg	NN	NR	NR

*GFR* glomerular filtration rate, *NN* not necessary, *NR* not recommended, *bid* 2 times daily

### 20. In an asymptomatic patient with recently diagnosed type 2 diabetes and HbA1c > 8.5%, combined pharmacological treatment for hyperglycemia consisting of metformin plus a second antihyperglycemic agent should be considered as first-line therapy. [IIa, C]

#### Summary of evidence

- This is an expert opinion-based recommendation, not based on published evidence. The majority of members from the Panel recommends to start combined therapy with metformin above HbA1c > 8.5% to avoid delaying the attainment of optimal glycemic control; all efforts should be made to prevent severe hyperglycemia in treatment-näive patients with type 2 diabetes.

### 21. In patients who do not achieve target HbA1c levels on monotherapy, any antidiabetic drug is potentially effective as an add-on option to metformin for glycemic control. There is no evidence of significant differences between classes of antidiabetic agents when used as second therapy added to metformin. [I, A] Pharmacological therapy to lower blood glucose in the patient with type 2 diabetes should be individualized on the basis of efficacy, mechanism of action, presence of comorbidities, risk of hypoglycemia, weight gain, adverse effects, and costs. [I, C]

#### Summary of evidence

- A meta-analysis [84] including 27 RCTs with 11,198 type 2 diabetes patients showed a similar HbA1c reduction between different classes of antidiabetic agents compared to placebo. The mixed-treatment comparison showed the following reductions in HbA1c: sulfonylureas, 0.79%; glinides, 0.65%; thiazolidinediones, 0.85%; α-glucosidase inhibitors, 0.64%; DPP-4 inhibitors, 0.78%; and GLP-1 analogues, 0.97%. Thiazolidinediones, sulfonylureas, and glinides were associated with mild weight gain, while GLP-1 analogues were associated with a significant decrease in body weight compared with placebo (−1.74 kg). There was no weight change associated with α-glucosidase inhibitors or DPP-4 inhibitors. Sulfonylureas and glinides were associated with an increased risk of hypoglycemia compared with placebo [84]. The choice of second antidiabetic agent should be based on efficacy, age, mechanism of action, risk of hypoglycemia, presence of comorbidities, life expectancy, weight gain or loss, adverse effects, and potential for cardiovascular protection [85].

*Outpatient treatment: third agent*

### 22. There is also no difference in HbA1c reduction when different classes of drugs are used as a third option for the treatment of type 2 diabetes. This panel recommends that any antidiabetic drug could be an option as a third agent for glycemic control, provided that the mechanism of action is not similar to that of agents already in use. [I, C]

#### Summary of evidence

- A meta-analysis of 18 RCTs (n-4535) evaluated the comparative efficacy of GLP-1 agonists, DPP-4 inhibitors, thiazolidinediones, and α-glucosidase inhibitors in reducing HbA1c, body weight, and causing severe hypoglycemia when a third drug was added to a metformin plus sulfonylurea regimen [86]. Despite limitations, as most of the studies were of short duration, with variable quality, and based on indirect comparisons, the meta-analysis showed that all antidiabetic classes were associated with significant reductions in HbA1c levels compared to placebo. The overall average reduction in Hba1c was −0.96% (thiazolidinediones, −1.15%; acarbose, −0.6%; GLP-1 agonists, −1.04%; DPP-4 inhibitors, −0.89%). There was no clear difference in efficacy between drug classes when adding a third agent to treatment of patients with type 2 diabetes who were already receiving metformin and a sulfonylurea [86].

### 23. Insulin therapy (with or without additional agents) should be considered any time in patients with type 2 diabetes who present persistently high blood glucose levels despite antidiabetic agent combinations, or in patients who are markedly symptomatic. [I, C]

#### Summary of evidence

- After at least 3 months using metformin plus a second antidiabetic agent, if the glycemic target is not reached, a third drug should be chosen, taking into account the established therapeutic target, age, patient limitations, and the attributes and side effects of each drug. Consider initiating insulin therapy (with or without additional agents) in patients with type 2 diabetes who remain markedly symptomatic (weight loss, ketosis, polyuria, or polydipsia) and/or exhibit elevated blood glucose levels or HbA1c [85].

### 24. Insulin is a safe option for glycemic control in type 2 diabetic patients treated with one or more antidiabetic agents who do not achieve HbA1c targets or who have typical symptoms of hyperglycemia, even in the presence of high cardiovascular risk. [I, A]

#### Summary of evidence

- The UKPDS [52] and ORIGIN [87] are randomized controlled trials that used human insulin and the insulin analogue glargine, respectively, in type 2 diabetes and evaluated long-term cardiovascular outcomes. The UKPDS revealed a 15% reduction in myocardial infarction and a 13% reduction in death among people with new-onset type 2 diabetes treated intensively with antidiabetic agents and insulin, as needed to attain an HbA1c of 7.0% vs. usual care. The mean follow up was of 10 years [52].
- In the ORIGIN study [87], participants were randomly assigned to insulin glargine added as an evening injection to their preexisting anti-hyperglycemic regimen or to standard care (treatment according to the investigator’s discretion in alignment with local guidelines). The study included 12,537 people, 88% of whom with type 2 diabetes, of which 59% had a previous cardiovascular event. After a mean follow up of 6.2 years, no differences were found between groups concerning the composite endpoint of nonfatal myocardial infarction, nonfatal stroke or cardiovascular death. These data indicate that basal insulin treatment (human insulin or insulin analogues) is safe in individuals with type 2 diabetes with or without pre-existing cardiovascular events.
- In the DEVOTE study (Efficacy and Safety of degludec versus glargine in type 2 diabetes), 7637 patients with type 2 DM were randomized to receive either insulin degludec or insulin glargine U100. The primary outcome (nonfatal myocardial infarction, nonfatal stroke and cardiovascular death) occurred in 8.5% of the patients treated with degludec and in 9.3% of the patients treated with glargine (hazard ratio = 0.91; *p* = non-significant). Patients treated with degludec experienced significant lower rates of severe hypoglycemia in comparison to the glargine U100 group (*p* < 0.001) [88].

*Cardiovascular risk*

### 25. In type 2 diabetic patients at VERY HIGH RISK (presence of clinical atherosclerotic disease, with previous cardiovascular events), the addition of an SGLT-2 inhibitor with demonstrated cardiovascular benefit can be useful to reduce cardiovascular risk, as it reduces the incidence of cardiovascular events and hospitalization due to heart failure in this population. [IIa, A]

#### Summary of evidence

- The EMPA-REG study of empagliflozin, an inhibitor of sodium glucose co-transporter-2 (SGLT2), evaluated 7020 high-risk patients with type 2 diabetes. After 3.1 years, empagliflozin therapy was associated with a 14% reduction in the composite primary outcome of CV mortality, nonfatal AMI, and nonfatal stroke (10.5% vs. 12.1%, *p* = 0.04; NNT 62), as well as a reduction in all-cause mortality (5.7% vs. 8.3%, *p* < 0.001; RRR −32%, NNT 38). There was also a reduction in cardiovascular mortality (3.7% vs. 5.9%, *p* < 0.001; RRR −38%, NNT 45) [89]. Interestingly, the HbA1c reduction with empagliflozin was modest (0.5%). The mechanisms by which the drug may have led to this significant result are still being studied.
- The CANVAS Program (Canagliflozin and Cardiovascular and Renal Events in Type 2 Diabetes) included 10,142 patients with type 2 DM, including individuals with established cardiovascular disease (secondary prevention) and patients at high risk for CV events (primary prevention). Patients were then randomized for Canagliflozin (100 mg and 300 mg) or placebo, and were followed for a mean of 188.2 weeks. Canagliflozin therapy was associated with a 14% reduction in the composite primary outcome of CV mortality, nonfatal AMI, and nonfatal stroke (occurring in 26.9 vs. 31.5 participants per 1000 patients-years). However, patients receiving canagliflozin experienced a significant increase in rates of amputation (6.3% vs. 3.4%; *p* < 0.001) and bone fractures (15.4% vs. 11.9%; *p* = 0.02) [90].
- Both EMPA-REG and CANVAS demonstrated a significant reduction in a secondary endpoint composed of hospitalization for heart failure and cardiovascular death.

### 26. In type 2 diabetic patients with clinical atherosclerotic disease (CLAD) (i.e., VERY HIGH-RISK patients), the addition of a GLP-1 analogue with demonstrated cardiovascular benefit may be useful to reduce cardiovascular risk, as it seems to decrease the incidence of cardiovascular events in this population. [IIa, A]

#### Summary of evidence

- The LEADER study of liraglutide, a GLP-1 analogue, assessed 9340 type 2 diabetes patients with high cardiovascular risk profile. After 3.8 years of follow-up, liraglutide was associated with a 13% reduction in the composite primary outcome of CV mortality, nonfatal AMI, and nonfatal stroke (13% vs. 14.9%, *p* = 0.01). There were reductions in cardiovascular mortality (4.7% vs. 6%, *p* = 0.007; RRR −22%) and all-cause mortality (8.2% vs. 9.6%; *p* = 0.02; RRR −15%). There was no reduction in the incidence of nonfatal AMI, nonfatal stroke, or hospitalization for heart failure [91].
- The SUSTAIN-6 trial analyzed 3297 patients with longstanding type 2 diabetes (mean disease duration 13.9 years) and established cardiovascular disease, chronic kidney disease, or both, on a standard care regimen, who were randomly assigned to receive once-weekly semaglutide (0.5 or 1.0 mg) or placebo for 104 weeks. At 2-year follow-up, there was a 26% reduction in the composite primary outcome or CV mortality, nonfatal AMI, and nonfatal stroke (6.6% vs. 8.9%, *p* = 0.02) [92]. Cardiovascular death was similar in the two groups (*p* = 0.92). Nonfatal stroke was the main composite primary outcome driver (1.6% vs. 2.7%, *p* = 0.04; RRR –39%). Diabetic retinopathy was more frequent in the semaglutide group (3%) than the placebo group (1.8%) (HR 1.76, 95% CI 1.11–2.78, *p* = 0.02). How much of this is due to a greater decrease in HbA1c still needs to be clarified (1% difference between semaglutide 1 mg and placebo).

### 27. In type 2 diabetic patients, at any level of risk of cardiovascular events, pioglitazone, DPP4 inhibitors, or GLP-1 analogues are safe and reasonable options to achieve glycemic control. [I, A]

#### Summary of evidence

- The use of pioglitazone in patients with long term type 2 diabetes and preexisting CV disease marginally reduced fatal and nonfatal myocardial infarction when compared to placebo (RRR −16%, 95% CI 0.72–0.98, *p* < 0.03). However, there was a twofold risk of hospitalization for heart failure and an increased risk of bone fractures in women, but no increase in mortality risk [93].
- Recently, several DPP-4 inhibitors and GLP-1 analogues have been evaluated for global CV safety and mortality outcomes in patients with type 2 diabetes at high risk of CV events.
- The TECOS (sitagliptin) study enrolled 14,671 patients with longstanding type 2 diabetes (mean disease duration 11.6 years), preexisting CV disease, and a mean baseline HbA1c of 7.2% [94].
- The SAVOR-TIMI 53 (saxagliptin) study examined 16,492 patients with longstanding type 2 diabetes (mean disease duration 10.3 years), preexisting CV disease or multiple risk factors, and an average baseline HbA1c of 8% [95].
- The EXAMINE (alogliptin) study evaluated 5380 patients with type 2 diabetes (mean disease duration 7.2 years) associated with acute coronary syndrome and average baseline HbA1c of 8% [96].
- The ELIXA (lixisenatide) study examined 6068 patients with type 2 diabetes (mean disease duration 9.4 years) associated with preexisting coronary artery disease with a recent hospital admission due to acute coronary syndrome and mean baseline HbA1c of 7.6% [97].
- Importantly, these studies were designed for noninferiority and demonstrated neutrality regarding global CV safety in patients with type 2 diabetes at high risk of CV events. Saxagliptin was associated with an unexpected increase in hospitalization for heart failure [94–97].

### 28. In type 2 diabetic patients at any level of cardiovascular risk, the use of sulfonylureas is safe and a reasonable option to achieve glycemic control. However, careful use of sulfonylureas is advocated because of a possible increased risk of hypoglycemia (especially in the elderly), as well as weight gain. [IIa, B]

#### Summary of evidence

- A meta-analysis of 47 RCTs (*n* = 37,650) evaluated the safety of the most frequently used sulfonylureas, in an attempt to elucidate conflicting data regarding the safety of this class of antidiabetics in terms of mortality and cardiovascular outcomes. The result showed that sulfonylureas were not associated with all-cause mortality (RR 12%, 95% CI 0.96–1.30) or cardiovascular mortality (RR 12%, 95% CI 0.87–1.42). Sulfonylureas were not associated with increased risk of myocardial infarction (RRR −8%, 95% CI 0.76–1.12) or stroke (RR 16%, 95% CI 0.81–1.66) [98].
- The Action in Diabetes and Vascular Disease: Preterax and Diamicron Modified Release Controlled Evaluation (ADVANCE) study and the ADVANCE-ON post-trial study were the largest (and with the highest CV risk population) ever conducted in patients with diabetes on sulfonylurea therapy in which cardiovascular outcomes were determined. The ADVANCE trial randomly assigned 11,140 patients with type 2 diabetes, of whom 32% had preexisting cardiovascular disease, to undergo either standard glucose control or intensive glucose control, defined as the use of gliclazide MR 60 mg/day to 120 mg/day, plus other drugs, to achieve an HbA1c value of 6.5% or less. After a median of 5 years of follow-up, the mean HbA1c level was lower in the intensive-control group (6.5%) than in the standard-control group (7.3%). Intensive control reduced the incidence of combined major macrovascular and microvascular events (18.1%, vs. 20.0% with standard control; HR 0.90, 95% CI 0.82–0.98, *p* = 0.01). This was due primarily to a reduction in the incidence of nephropathy (4.1% vs. 5.2%; HR 0.79, 95% CI 0.66–0.93, *p* = 0.006). Importantly, there was no increase in death from all causes (*p* = 0.91) nor from cardiovascular causes (*p* = 0.63). The ADVANCE-ON study invited 8944 surviving participants from the ADVANCE study to a 6-year post-trial study, defining death from any cause and major macrovascular events as primary endpoints. Between-group differences in HbA1c levels during the trial were no longer evident. No differences were observed in risk of death from any cause (*p*=) or major macrovascular events between the intensive-control group and the standard-control group (HR 1.00, 95% CI 0.92–1.08 and HR 1.00, 95% CI 0.92–1.08 respectively).

## Module 5: Management of dyslipidemia

### 29. In patients with diabetes at VERY HIGH RISK, the recommended lipid target is to reduce LDL-c to a level below 50 **mg/dL or non-HDL-c to a level below 80** **mg/dL (Table** **7****). For patients not on statin treatment, at any baseline LDL-c level, an initial reduction in LDL-c or in non-HDL-c of more than 50% from baseline is recommended. [I, A]**

**Table 7:**
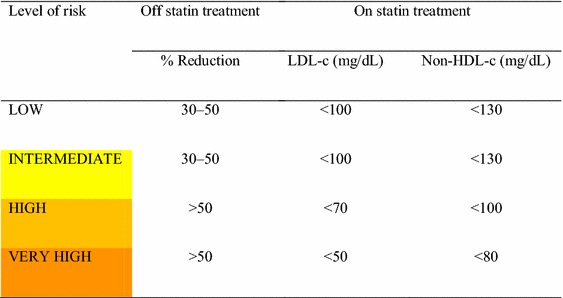
Cholesterol targets in patients with diabetes

### 30. If, in VERY HIGH-risk patients, after 3 months, targets are not met, treatment must be intensified. [I, C]

#### Summary of evidence

- Two double-blind, controlled, randomized clinical trials have demonstrated that reducing the levels of LDL-c cholesterol to below (or near) 50 mg/dL is associated with a significant reduction in the incidence of major cardiovascular events. In the FOURIER trial [99], 27,564 patients with atherosclerotic cardiovascular disease and under statin therapy were randomized to placebo or evolocumab. Patients randomized to evolocumab had their LDL-c levels reduced to 30 mg/dL and had a significant reduction in major cardiovascular events (9.8% in the evolocumab group vs 11.3% in the placebo group, hazard ratio 0.85, *p* < 0.001). In the IMPROVE-IT trial [100], 18,144 patients who had been hospitalized for acute coronary syndrome in the preceding 10 days were randomized to simvastatin or simvastatin + ezetimibe. Patients randomized to simvastatin plus ezetimibe had their LDL-c levels reduced to 53.7 mg/dL and experienced a significant reduction in cardiovascular events (32.7% for the simvastatin/ezetimibe group vs 34.7% for the simvastatin group, hazard ratio 0.936, *p* = 0.016).
- Statins have largely been proven to reduce the risk of cardiovascular events in patients with diabetes with a previous history of vascular events. A meta-analysis of 14 trials including 18,686 patients with diabetes concluded that statin treatment reduces the incidence of vascular events proportionately by 20% for each 39 mg/dL reduction in LDL-c in 5 years, with a similar reduction for major coronary events, stroke, and need for revascularization [101].
- In a meta-analysis of individual data from 8 statin RCTs [102] including 38,153 patients allocated into statin therapy, in which lipids and apolipoproteins were determined at baseline and after 1 year of follow-up, a total of 6286 major cardiovascular events were observed in 5387 study participants. Patients with LDL-c below 50 mg/dL were at significantly lower risk than patients with increased levels of LDL-c. The risk category was proportionally lower as the level of LDL-c decreased. Compared with patients whose LDL-c was >175 mg/dL, those who reached an LDL-c of 75–100 mg/dL, 50–75 mg/dL, and <50 mg/dL respectively had progressively lower adjusted HRs of 0.56 (95% CI 0.46–0.67), 0.51 (95% CI 0.42–0.62), and 0.44 (95% CI 0.35–0.55) for major cardiovascular events. Similar associations were observed for non-HDL-c and apolipoprotein B. LDL-c limits may be transferred to non-HDL-c limits by adding 30 mg/dL [103].
- Non-HDL-c is calculated by subtracting HDL-c from total cholesterol. This measure is not affected by triglyceride concentration and is better than calculated LDL-c in patients with increased plasma triglyceride concentrations.

### 31. Patients with diabetes in the VERY HIGH-RISK category should initiate statins as soon as possible at the highest tolerable dose (Table 8) to meet cholesterol targets (Table 7). The lipid profile should be reviewed every 1–3 months. If targets are not met, intensification of treatment is advised, either by switching to a more potent statin, increasing statin dose, adding ezetimibe, and/or improving lifestyle modifications. [I, A]

#### Summary of evidence

- A pre-specified subgroup analysis of the treat to new targets (TNT) study [104], which included 1501 patients with diabetes and coronary artery disease, compared the impact of atorvastatin 80 mg vs. 10 mg on cardiovascular outcomes during 4.9 years. The study showed a significant reduction in any cardiovascular event and stroke in the 80 mg arm. The lower LDL-c attained with the highest dose showed additional benefit.

**Table 8:** Mean expected % of LDL-c reduction with statin use

Statin	Mean expected LDL-c reduction (%)		
	<30 (mg)	30–50 (mg)	≥50
Simvastatin	10	20–40	40 mg + ezetimibe
Pravastatin	10–20	40–80	–
Fluvastatin	20–40	80	–
Atorvastatin	–	10–20	40–80 mg
Rosuvastatin	–	5–10	20–40 mg
Pitavastatin	1	2–4	–
Lovastatin	20	40	–
- A meta-analysis of five randomized trials [105] (39,612 subjects with prior vascular disease, 5639 [14%] with diabetes) compared intensive vs. moderate statin treatments. Mean follow-up was of 5.1 years. Intensive treatment was defined as a reduction in LDL-c of 20 mg/dL beyond the result obtained by moderate treatment with the use of higher-potency statins. The results showed a 15% further reduction in major vascular events (95% CI 11–18, *p* < 0.0001), 13% in coronary death (95% CI 7–19, *p* < 0.0001), 19% in coronary revascularization (95% CI 15–24), *p* < 0.0001) and 16% in stroke (95% CI 0.74–0.95, *p* = 0.005). Moderate treatment promoted a 30% decrease in cardiovascular events compared to placebo. Intensive treatment promoted a 20% reduction in cardiovascular events beyond moderate treatment. Thus, the overall reduction in events with intensive treatment compared to moderate treatment was 50%.
- Treatment goals, even with the highest tolerated statin dose, may not be reached by patients with dyslipidemia, particularly those with established CVD, DM, or asymptomatic high-risk individuals. In such cases, combination treatment may be needed. However, the only combination with evidence of clinical benefit (one large RCT) is that of a statin and with ezetimibe [100]. Based on the relatively limited evidence, the ESC/EAS 2016 panel recommends restricted use of this combination in patients at high or very high risk of CVD [103].

### 32. The use of PCSK9 inhibitors may be considered in VERY HIGH-RISK patients who do not meet LDL-c targets despite high intensity statin use. The decision to use PCSK9 inhibitors, however, must be carefully evaluated through cost-benefit analysis. [IIa, B]

- Monoclonal antibody inhibitors of proprotein convertase subtilisin–kexin type 9 serine protease (PCSK9), a protein that regulates the recycling of LDL receptors, have recently been approved by the FDA, EMEA, and ANVISA for primary prevention in patients with hetero- and homozygous familial hypercholesterolemia or as secondary prevention in patients with CLAD who require additional LDL-c–lowering therapy. This class of drugs meets a large unmet need for more aggressive lipid-lowering therapy beyond statins in an attempt to further reduce residual risk in many persons with clinical CLAD and diabetes. When added to maximal statin therapy, these once- or twice-monthly injectable agents reduce LDL-c by approximately 60%, and have favorable effects on other lipids [106–112]. In post hoc cardiovascular safety analyses of alirocumab and evolocumab added to statins with or without other lipid-lowering therapies, mean LDL-c levels of 48 mg/dL were associated with statistically significant relative risk reductions of 48–53% in major CLAD events [107, 108]. Furthermore, a subgroup analysis of patients with diabetes taking alirocumab demonstrated that a 59% LDL-c reduction was associated with a CLAD event relative risk reduction trend of 42% [113].
- The FOURIER study [99] was a randomized, double-blind, placebo controlled trial which evaluated whether the PCSK9 inhibitor evolocumab associated with a statin could reduce cardiovascular risk vs. statin therapy alone in patients with clinically evident atherosclerotic cardiovascular disease and LDL-c levels of 70 mg/dL. After 48 weeks, evolocumab reduced LDL-c from a baseline of baseline of 92–30 mg/dL and met its primary composite endpoint, reducing cardiovascular death, nonfatal myocardial infarction (MI), nonfatal stroke, hospitalization for unstable angina or coronary revascularization in 15% (*p* < 0.001). The key secondary composite endpoint (cardiovascular death, nonfatal MI or nonfatal stroke) was also reduced in 20% (*p* < 0.001). No new safety issues were observed.
- In the GLAGOV study [114], in patients with angiographic coronary disease treated with statins, addition of evolocumab, compared with placebo, resulted in a greater decrease in PAV (percent atheroma volume) after 76 weeks of treatment. The evolocumab group achieved lower mean, time-weighted LDL-c levels (93.0 vs. 36.6 mg/dL; difference, −56.5 mg/dL [95% CI −59.7 to −53.4], *p* < 0.001). The primary efficacy parameter, PAV, increased 0.05% with placebo and decreased 0.95% with evolocumab (difference, −1.0% [95% CI −1.8 to −0.64%], *p* < 0.001). The secondary efficacy parameter, normalized TAV (total atheroma volume), decreased 0.9 mm^3^ with placebo and 5.8 mm^3^ with evolocumab (difference, −4.9 mm^3^ [95% CI −7.3 to −2.5]; *p* < 0.001). Evolocumab induced plaque regression in a greater percentage of patients than placebo (64.3% vs. 47.3%; difference, 17.0% [95% CI 10.4–23.6], *p* < 0.001 for PAV and 61.5% vs. 48.9%; difference, 12.5% [95% CI 5.9–19.2%], *p* < 0.001 for TAV) [114].

### 33. In patients with diabetes at VERY HIGH RISK (Tables 2, 5) with a recent acute coronary syndrome, lipid profile should be determined in the first 12–24 h of hospitalization to define baseline levels. Subsequently, statin treatment should be started at the highest tolerable doses, as soon as possible, for 3 months, independently of lipid levels. At that time, lipid profile should be reassessed to check for target achievement. [I, B]

#### Summary of evidence

- The double-blind, randomized IMPROVE IT trial [100] studied 18,144 patients who had been hospitalized for acute coronary syndrome within the preceding 10 days and had LDL-c levels of 50–100 mg/dL while on lipid-lowering therapy or 50–125 mg/dL if they were not receiving lipid-lowering therapy. The combination of simvastatin (40 mg) and ezetimibe (10 mg) (simvastatin–ezetimibe) was compared with simvastatin (40 mg) and placebo. The primary outcome was the composite of cardiovascular death, nonfatal myocardial infarction and unstable angina requiring re-hospitalization, coronary revascularization, or nonfatal stroke. The median follow-up was 6 years. The median time-weighted mean LDL-c level during the study was 53.7 mg/dL in the simvastatin–ezetimibe group, as compared with 69.5 mg/dL in the simvastatin alone group (*p* < 0.001). The event rate for the primary endpoint at 7 years was 32.7% in the simvastatin–ezetimibe group, as compared with 34.7% in the simvastatin-alone group, with an absolute risk difference of 2.0% (HR 0.936, 95% CI 0.89–0.99, *p* = 0.016). Thus, vigorous reduction of LDL-c in the early phases of acute coronary syndrome resulted in improved cardiovascular outcomes and should be recommended. In addition, subgroup analysis revealed the greatest benefit in patients with diabetes, with a 15% reduction in the primary endpoint and a NNT = 18 [100].

**34. In patients with diabetes at HIGH RISK (Tables** **2**, **3**, **4** **), LDL-c should be maintained below 70** **mg/dL and/or non-HDL-c below 100** **mg/dL. [I, A]**

### 35. Alternatively, in HIGH-RISK diabetic patients not using statins, an initial reduction of >50% in LDL-c or in non-HDL-c is recommended. If, after 3 months, targets are not met (LDL-c < 70 mg/dL or non-HDL-c < 100 mg/dL), treatment should be intensified. [I, C]

#### Summary of evidence

- The Collaborative Atorvastatin Diabetes Study (CARDS) [115], which was terminated early for efficacy, assessed 2838 patients with diabetes without coronary artery disease (age 40–75 years) and at least 1 additional risk factor (microalbuminuria, retinopathy, hypertension, or smoking). Patients were randomized to atorvastatin 10 mg or placebo during a mean follow-up of 3.9 years, a composite of acute coronary events, coronary revascularization, or stroke as primary outcome. Atorvastatin 10 mg was associated with risk reduction of 37% (95% CI −52 to −17, *p* = 0.001) in the primary endpoint, reduction of 32% (95% CI −45 to −15, *p* = 0.001) in stroke risk, and a trend toward 27% reduction in total mortality (95% CI −48 to 1.0, *p* = 0.059). The CARDS estimated that one event is avoided for every 27 patients treated for 4 years.
- In the MRC/BHF Heart Protection Study (HPS) substudy [116], 5963 individuals with diabetes (age 40–80 years) were randomized to simvastatin 40 mg or placebo. A pre-specified subgroup analysis was performed for the outcomes of fatal and nonfatal acute myocardial infarction (AMI) and first vascular event (major coronary event, stroke, or revascularization). Simvastatin 40 mg reduced these outcomes by 33% (95% CI 17–46, *p* < 0.0003), regardless of baseline LDL-c level. The absolute reduction of cardiovascular disease risk in patients with diabetes without coronary artery disease was similar for the HPS and the CARDS studies. This confirms the benefit of statins for primary prevention in high-risk patients with diabetes.
- In the pre-specified subgroup analysis of the treat to new targets (TNT) study [104], with 1501 patients with diabetes and coronary artery disease, intensive treatment with atorvastatin 80 mg was associated with a significant reduction in any cardiovascular events and stroke compared with atorvastatin 10 mg in a 4.9-year follow-up. Patients taking 10 mg achieved an average level of LDL-c of 96 mg/dL, while those using 80 mg/day achieved 77 mg/dL. Thus, the attainment of a target LDL-c below 70 mg/dL showed additional benefit.
- A systematic search and meta-analysis including 11 trials was performed to evaluate the impact of statin therapy on carotid intima media thickening (CIMT) progression. Statin therapy was found to slow the progression of carotid atherosclerosis, indicating benefits at the subclinical stage of the disease process [117].

### 36. In patients with diabetes at HIGH RISK, with either stratifying factors (Table **3****) or confirmed subclinical atherosclerosis (Table** **4****), it is highly recommended to start statin therapy (Table** **8****) to meet targets (Table** **7****). [I, A]**

### 37. If, after 3 months, LDL-c or non-HDL-c is not at the defined target, intensification of therapy should be considered. [IIa, B]

#### Summary of evidence

- In the CTT meta-analysis [105] moderate treatment promoted a 30% decrease in cardiovascular events compared to placebo. Intensive treatment promoted a 20% reduction in cardiovascular events beyond moderate treatment. Thus, there was an overall 50% reduction in events with intensive treatment compared to moderate treatment. Despite the indirect evidence provided by subgroup analysis of diabetic patients in the meta-analysis, the absence of heterogeneity makes these results applicable to patients with DM in primary prevention.

### 38. In patients with diabetes at LOW-INTERMEDIATE RISK, LDL-c levels should be lowered and maintained below 100 mg/dL and non-HDL-c levels should be lowered and maintained below 130 mg/dL (Table 7). [I, B]

#### Summary of evidence

- In a meta-analysis of 14 trials including 18,686 individuals with diabetes, statin therapy reduced all-cause mortality and vascular mortality, and the reduction in vascular events was proportional to the LDL-c reduction. The proportional effects of statins in diabetic patients were similar irrespective of prior history of vascular disease or other baseline clinical conditions [101].

### 39. Statins are initially optional for LOW RISK patients, but should be considered in INTERMEDIATE RISK patients (Table 9), if LDL-c and non-HDL-c are above the targets (Table 7). Lipid profile should be re-checked periodically to ensure that LDL-c level is below 100 mg/dL. Intensification of treatment is needed if targets are not met. [IIa, C]

#### Summary of evidence

- The TRIALIST meta-analysis [118] compared the effects of lowering cholesterol with statins on the incidence of cardiovascular events in a low-risk population. The meta-analysis included 22 statin vs. control trials (*n* = 134,537) with mean follow-up duration of 4.8 years, and five more vs. less statin trials (*n* = 39,612) with 5.1 years of follow-up. Participants were separated into five categories of baseline 5-year major vascular event risk on control therapy (<5, ≥5 to <10, ≥10 to <20, ≥20 to <30, ≥30%), with estimation of the rate ratio (RR) per 1.0-mmol/L LDL-c reduction in each category. Reduction of LDL cholesterol with a statin reduced the risk of major vascular events (RR 0.79, 95% CI 0.77–0.81, per 1.0 mmol/L reduction), irrespective of age, sex, baseline LDL-c, or previous vascular disease, and of vascular and all-cause mortality. The proportional reduction in major vascular events was at least as great in the two lowest risk categories as in the higher risk categories. This reflected significant reductions in major coronary events in the two lowest risk categories. In individuals with 5-year risk of major vascular events <10%, each 1-mmol/L reduction in LDL-c produced an absolute reduction in major vascular events of about 11 per 1000 over 5 years. These data indicate that low-risk populations also benefit from lowering cholesterol with statins.

**Table 9:**
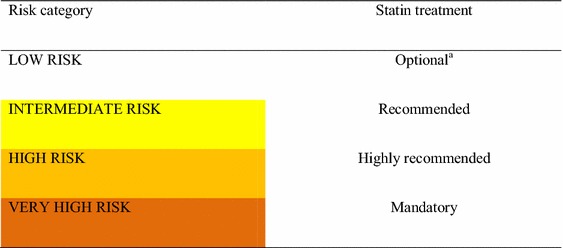
Recommendation for statin treatment according to cardiovascular risk category in diabetes

^a^Optional means that non-pharmacological (lifestyle) measures are acceptable, provided that an LDL-c target <100 mg/dL is attained and maintained. For patients with LDL-c >160 mg/dL, statins are advisable at any risk category

### 40. It is recommended that patients with diabetes and LDL-c > 190 mg/dL be investigated for familiar hypercholesterolemia (FH). [I, C]

#### Summary of evidence

- The diagnosis of FH in patients with diabetes should be always considered and further investigated when an LDL-c level > 190 mg/dL is found [106]. LDL-c > 250 mg/dL in a patient aged 30 or older, LDL-c > 220 mg/dL in patients aged 20–29, and LDL-c > 190 mg/dL in patients under age 20 yields approximately 80% probability of FH in the setting of general population screening [107].

### 41. It is recommended that patients with diabetes and chronic kidney failure who are on dialysis, without CLAD (Table 5), do NOT initiate use of statins, since there is no evidence of benefit in this population and, in fact, the risk of stroke may increase. [III, A] However, in patients with chronic renal failure who were already on statin therapy before initiation of dialysis, withdrawal of statins is not recommended. [III, A]

#### Summary of evidence

- In the 4D (die deutsche diabetes dialyze) study [119], 1255 patients with type 2 diabetes on hemodialysis were evaluated. They were randomized to atorvastatin 20 mg or placebo and followed up for 4 years. The primary endpoint was a composite of death from cardiac causes, nonfatal myocardial infarction, and stroke. A 42% reduction in LDL-c was observed in patients on atorvastatin, with no reduction in the primary outcome. The risk of stroke was also increased in this group.
- The study to evaluate the use of rosuvastatin in subjects on regular hemodialysis (AURORA) study [120] included 2776 hemodialysis patients (aged 50–80, 27.9% with diabetes) treated with rosuvastatin 10 mg/day or placebo during a mean of 3.8 years. The primary outcome was a composite of nonfatal myocardial infarction, nonfatal stroke, and cardiovascular death. There was a 43% reduction in LDL-c in the intervention group, but no differences in the primary outcome were observed between groups.
- Regarding patients with chronic renal disease but not on hemodialysis, the Pravastatin Pooling Project database made a combined analysis of results of three randomized trials of pravastatin 40 mg vs. placebo [121], including 19,700 patients with chronic renal insufficiency (estimated GFR 60–30 mL/min/1.73 m^2^). Significant benefit of treatment was detected in reducing the primary endpoint of myocardial infarction, coronary death, or percutaneous revascularization and total mortality in this group of patients.
- The SHARP trial aimed to assess the efficacy and safety of the combination of simvastatin plus ezetimibe in people with moderate-to-severe kidney disease. This randomized, double-blind trial included 9270 patients with chronic kidney disease (3023 on dialysis and 6247 not on dialysis) with no known history of myocardial infarction or coronary revascularization. Patients were randomly assigned to simvastatin 20 mg plus ezetimibe 10 mg daily versus matching placebo. The key pre-specified outcome was first major atherosclerotic event (nonfatal myocardial infarction or coronary death, non-hemorrhagic stroke, or any arterial revascularization procedure). All analyses were by intention to treat. A total of 4650 patients were assigned to receive simvastatin plus ezetimibe, and 4620 to placebo. Allocation to simvastatin plus ezetimibe yielded an average LDL cholesterol difference of 33 mg/dL (SE 0.02, with about two-thirds of the sample adherent) during a median follow-up of 4.9 years, and produced a 17% proportional reduction in major atherosclerotic events (526 [11.3%] simvastatin plus ezetimibe vs. 619 [13.4%] placebo; rate ratio [RR] 0.83, 95% CI 0.74–0.94, log-rank *p* = 0.0021). Patients allocated to simvastatin plus ezetimibe did not differ with respect to nonfatal myocardial infarction or death from coronary heart disease (213 [4.6%] vs. 230 [5.0%]; RR 0.92, 95% CI 0.76–1.11, *p* = 0.37), and there were significant reductions in non-hemorrhagic stroke (131 [2.8%] vs. 174 [3.8%]; RR 0.75, 95% CI 0.60–0.94, *p* = 0.01) and arterial revascularization procedures (284 [6.1%] vs. 352 [7.6%]; RR 0.79, 95% CI 0.68–0.93, *p* = 0.0036). Adjustment for subgroup-specific reductions in LDL-c did not reveal evidence of differences between the proportional effects on major atherosclerotic events and the summary rate ratio in any subgroup examined, and, in particular, in patients on dialysis vs. those who were not on dialysis. The study concluded that reduction of LDL cholesterol with simvastatin 20 mg plus ezetimibe 10 mg daily safely reduced the incidence of major atherosclerotic events in patients with advanced chronic kidney disease [122].
- A sub-analysis of the treating to new targets study investigated how intensive lipid lowering with 80 mg of atorvastatin affects renal function when compared with 10 mg in patients with coronary heart disease. A total of 10,001 patients with coronary heart disease and LDL-c levels < 130 mg/dL were randomly assigned to double-blind therapy with 10 or 80 mg/d atorvastatin. Estimated GFR using the modification of diet in renal disease equation was compared at baseline and at the end of follow-up in 9656 participants with complete renal data. The expected 5-year decline in renal function was not observed. However, estimated GFR improved in both treatment groups, but was significantly greater with 80 mg than with 10 mg, suggesting this benefit may be dosage-related [123].
- Another sub-analysis of the TNT study investigated the effects of intensive lipid lowering with atorvastatin in patients with coronary heart disease (CHD) with and without preexisting chronic kidney disease (CKD). The study concluded that aggressive lipid lowering with atorvastatin 80 mg was both safe and effective in reducing excess cardiovascular events in a high-risk population with CKD and CHD [124].

### 42. In patients with diabetes and class III–IV heart failure, initiation of statin therapy is not recommended because there is no clear evidence of benefit in this group. [III, A]

#### Summary of evidence

- The effect of rosuvastatin in patients with chronic heart failure (GISSI-HF) randomized, multicenter clinical trial evaluated rosuvastatin 10 mg/day compared to placebo in 2285 patients with heart failure due to any cause or condition (New York Heart Association classes II–IV); 26% also had diabetes. There was no benefit in the outcomes of interest (death and hospitalization for cardiovascular causes) [125].
- The controlled rosuvastatin multinational trial in heart failure (CORONA) randomized study compared the use of rosuvastatin 10 mg versus placebo in 5011 patients aged >60 years with class II–IV heart failure of ischemic etiology (including 29% with diabetes). The primary endpoint was a composite of cardiovascular death, acute nonfatal MI, and nonfatal stroke during 36 months. Despite a 45% reduction in LDL-c, there was no significant between-group difference in the primary endpoint. The results were extensive to patients with diabetes in the subgroup analysis, due to low heterogeneity [126].
- A retrospective analysis of the CORONA trial compared 10 mg rosuvastatin daily with placebo in patients with ischemic systolic heart failure according to baseline high sensitivity-C reactive protein (hs-CRP) <2.0 mg/L (placebo, n = 779; rosuvastatin, *n* = 777) or ≥2.0 mg/L (placebo, *n* = 1694; rosuvastatin, *n* = 1711). The primary outcome was cardiovascular death, myocardial infarction, or stroke. The study demonstrated a significant interaction between hs-CRP and the effect of rosuvastatin for most endpoints, whereby rosuvastatin treatment was associated with better outcomes in patients with hs-CRP ≥ 2.0 mg/L [127]. In addition, patients with heart failure due to ischemic heart disease who had NT-proBNP values <103 pmol/L (868 pg/mL) had the best prognosis and, if assigned to rosuvastatin rather than placebo, had a greater reduction in the primary endpoint (HR 0.65, 95% CI 0.47–0.88) than patients in the other tertiles (heterogeneity test, *p* = 0.0192). This reflected fewer atherothrombotic events and sudden deaths in the active group, and may show a benefit from rosuvastatin use [128].

### 43. In the patient with diabetes and mild to moderate hypertriglyceridemia (TG 150–400 mg/dL), the combination of a statin and a fibrate is not usually recommended for reduction of cardiovascular risk. However, in the specific situation of a patient with triglycerides >204 mg/dL and HDL-c < 34 mg/dL, the combination of fenofibrate and a statin can be considered when lifestyle modifications have failed. [IIa, B]

#### Summary of evidence

- The pre-specified subgroup analysis of patients with diabetes from the ACCORD-LIPID (action to control cardiovascular risk in diabetes-lipids arm) study [129], comparing micronized fenofibrate 160 mg plus simvastatin 20–40 mg versus simvastatin 20–40 mg alone plus fenofibrate placebo, showed no reduction in the primary outcome. However, there was benefit in the pre-specified subgroup analysis of patients with triglycerides >204 mg/dL and HDL-c < 34 mg/dL.
- The FIELD (fenofibrate intervention and event lowering in diabetes) multinational RCT, randomized 9795 individuals with type 2 diabetes mellitus (aged 50–75 years, 2131 with previous cardiovascular disease and 7664 without) not on statin treatment at study enrollment to receive micronized fenofibrate 200 mg daily (*n* = 4895) or matching placebo (*n* = 4900) for 5 years of follow up. The primary outcome was coronary heart disease death or nonfatal myocardial infarction. The pre-specified outcome for subgroup analyses was total cardiovascular events (the composite of cardiovascular death, myocardial infarction, stroke, and coronary and carotid revascularization). Fenofibrate did not reduce risk of the primary outcome. However, it reduced the secondary pre-specified outcome of total cardiovascular events, due to fewer nonfatal myocardial infarctions and revascularizations [130].

## Module 6. Management of hypertension

*Targets*

### 44. In patients with diabetes without clinical atherosclerotic disease (CLAD), blood pressure targets of a systolic blood pressure (SBP) < 130 mmHg and a diastolic blood pressure (DBP) < 80 mmHg may be reasonable, if well tolerated by the patient. [IIb, B]

#### Summary of evidence

- In the ACCORD study [131] of 4733 diabetic patients, randomization to an SBP target <120 mmHg vs. <140 mmHg could not reduce significantly the risk of the study’s primary outcome (HR 0.88, 95% CI 0.73–1.06, *p* = 0.20). Thus, the results of the study do not support the recommendations for stricter BP targets in this patient population. The mean SBP achieved in the first year of treatment in this trial were 119.3 mmHg for the <120 mmHg arm and 133.5 mmHg for the 140 mmHg arm, respectively. However, in the SBP < 120 mmHg arm, there was a 41% reduction in risk of stroke (HR 0.59, 95% CI 0.39–0.89, *p* = 0.01) with a low incidence of adverse events.
- The ACCORD BP study used a 2 × 2 factorial design, which also included comparisons of standard or intensive glycemic targets combined with intensive or standard blood pressure control in the same trial. A secondary pre-specified analysis [132] showed that, when combining intensive glycemic control with intensive blood pressure control, the rate of major CVD outcomes was significantly lowered when compared with combined standard BP and standard glycemic control.
- In a network meta-analysis including 42 clinical trials with random allocation into anti-hypertensive medication, control, or treatment target, a total of 144,220 individuals were compared in different strata of systolic blood pressure (SBP) to define the best target to reduce cardiovascular disease and all-cause mortality. In 30 trials, patients with type 2 diabetes were included. Patients were analyzed according to their mean achieved SBP in nine strata: 120–124; 125–129; 130–134; 135–139; 140–144; 145–149; 150–154; 154–159; and >160 mmHg. There were linear associations between mean achieved SBP and the risk of cardiovascular disease and mortality, with the lowest risk in the lowest stratum (120–124 mmHg). Individuals who achieved SBP 120–124 mmHg had a HR for all-cause mortality of 0.73 (95% CI 0.58–0.93) compared to those in the SBP 130–134 mmHg stratum: HR 0.59 (95% CI 0.45–0.77). Thus, reducing SBP levels to below 130 mmHg is associated with significant reductions in cardiovascular disease and in all-cause mortality [133].

### 45. In patients with established coronary heart disease (CLAD), it is not recommended to reduce blood pressure below 120/70 mmHg. [III, B]

#### Summary of evidence

- Because coronary perfusion occurs mainly during diastole, patients with coronary artery disease (CAD) could be at increased risk for coronary events if DBP falls below critical levels. A secondary analysis of data from the International Verapamil-Trandolapril Study (INVEST), including 22,576 patients with hypertension and CAD, determined whether low blood pressure could be associated with excess mortality and morbidity in this population. The analysis found a progressive increase for the risk for the primary outcome, all-cause death, and MI, but not stroke, with low DBP. The authors concluded that excessive reduction in diastolic pressure should be avoided in patients with CAD who are being treated for hypertension [134].
- Data from 22,672 patients with stable coronary artery disease from 45 countries enrolled in the CLARIFY registry and treated for hypertension were analyzed to ascertain whether a relationship exists between achieved blood pressure rates and cardiovascular events. SBP and DBP before each event were averaged and categorized into 10-mmHg increments. The primary outcome was a composite of cardiovascular death, MI, or stroke. Hazard ratios (HR) were estimated with multivariable adjusted Cox proportional hazards models, using 120–129 mmHg SBP and 70–79 mmHg DBP subgroups as references. The study concluded that, in patients with hypertension and coronary artery disease from routine clinical practice, SBP < 120 mmHg and DBP < 70 mmHg were each associated with adverse cardiovascular outcomes, including mortality, supporting the existence of a J-curve phenomenon. Thus, caution is advised in the use of antihypertensive treatment in patients with coronary artery disease [135].
- The ARIC (Atherosclerosis Risk In Communities) cohort of 11,565 adults analyzed associations between DBP and high-sensitivity cardiac troponin T (hs-cTnT) levels, as well as prospective associations between DBP and CV events. Compared with persons who had DBP 80–89 mmHg at baseline (ARIC visit 2), the adjusted odds ratio of having hs-cTnT ≥ 14 ng/L at that visit was 2.2 and 1.5 in those with DBP < 60 mmHg and 60–69 mmHg, respectively. Low DBP at baseline was also independently associated with progressive myocardial damage on the basis of estimated annual change in hs-cTnT over the 6 years between ARIC visits 2 and 4. In addition, compared with a DBP of 80–89 mm Hg, a DBP < 60 mmHg was associated with incident CHD and mortality, but not with stroke. The DBP and incident CHD association was strongest with baseline hs-cTnT ≥ 14 ng/L (*p* value for interaction <0.001). Associations of low DBP with prevalent hs-cTnT and incident CHD were most pronounced among patients with baseline SBP ≥ 120 mmHg. The study concluded that, among adults with an SBP ≥ 120 mmHg (and, thus, elevated pulse pressure), low DBP was associated with subclinical myocardial damage and CHD events. When titrating treatment to SBP < 140 mmHg, it may be prudent to ensure that DBP levels do not fall below 70 mmHg and, particularly, not below 60 mmHg [136].

### 46. In patients with diabetes aged 80 years or older, a systolic blood pressure target <150 mmHg is reasonable. [IIa, B]

#### Summary of evidence

- In the hypertensive elderly (age ≥ 80 years), there is no evidence of benefits deriving from BP levels <140 mmHg, but there is an increased likelihood of adverse effects. The HYVET Study supports the recommendation of a BP target <150/90 mmHg, with a reduction in the risk of stroke and HF [137, 138]. The presence of isolated systolic hypertension (ISH) requires care regarding excessive reduction in DBP, which should be maintained over 60 mmHg or even over 65 mmHg in the presence of CAD [139].
- The SPRINT study reported a 24% reduction in the risk of the study’s primary outcome in elderly patients (age ≥75 years) allocated to the more intense BP treatment arm (mean SBP achieved, 123.4 mmHg) as compared to the group of standard SBP reduction (mean BP achieved, 134.8 mmHg). This occurred regardless of degree of fragility, with no increase in the number of adverse events in relation to the rest of the study population [140]. That suggests that BP targets for the elderly should be defined in the same as for other adults. It should be noted, however, that BP reduction should be performed carefully, considering comorbidities and the use of multiple medications.

### 47. In patients with stage III hypertension (defined as blood pressure ≥180/110 mmHg), the initial target blood pressure should be <140/90 mmHg. [I, A]

#### Summary of evidence

- In a meta-analysis, Thomopoulos et al. investigated if treatment to lower blood pressure benefits all grades of hypertension and determined the target BP levels to maximize outcome reduction. Significant outcome reductions were found independently of hypertension grade. No trend was observed toward changes in risk ratio with increasing baseline BP. In 32 RCTs (128,232 individuals), relative and absolute outcome reductions were significant for the SBP differences across 150 and 140 mmHg cutoffs. Below 130 mmHg, only stroke and all-cause mortality were significantly reduced. There was a significant trend toward greater absolute outcome reduction with lower SBP cutoffs. In 29 RCTs (107,665 individuals), outcomes were significantly reduced across DBP cutoffs of 90 and 80 mmHg. After excluding RCTs with baseline DBP <90  mmHg, only stroke reduction was significant at achieved DBP <80 mmHg. In conclusion, meta-analyses favor BP-lowering treatment in all grades of hypertension, at low-to-moderate risk, and lowering SBP/DBP to less than 140/90 mmHg. Achieving <130/80 mmHg appears safe, but only adds further reduction in stroke [141].

### 48. In patients with diabetes and increased albuminuria (>30 mg/g of creatinine), it is recommended that systolic blood pressure and diastolic blood pressure targets should be <130 and <80 mmHg respectively. [I, A]

#### Summary of evidence

- In the ADVANCE randomized clinical trial [142], 11,140 patients with type 2 diabetes and hypertension were randomized to receive with a fixed combination of perindopril and indapamide or matching placebo, in addition to current therapy. The primary endpoints were composites of major macrovascular and microvascular events (death from cardiovascular disease, nonfatal stroke, or nonfatal myocardial infarction) and new or worsening renal or diabetic eye disease. Analysis was by intention-to-treat. The macrovascular and microvascular composites were analyzed jointly and separately. Patients assigned to active therapy had a mean SBP reduction of 5.6 mmHg and a mean DBP reduction of 2.2 mmHg compared to the placebo arm. The relative risk of a major macrovascular or microvascular event was reduced by 9% (861 [15.5%] active vs. 938 [16.8%] placebo; HR 0.91, 95% CI 0.83–1.00, *p* = 0.04). The separate reductions in macrovascular and microvascular events were similar, but not independently significant (macrovascular: 0.92, 0.81–1.04, *p* = 0.16; microvascular: 0.91, 0.80–1.04, *p* = 0.16).
- In the IRMA-2 multinational, double-blind RCT, 590 hypertensive patients with type 2 diabetes and microalbuminuria were enrolled to receive irbesartan 150 mg daily or 300 mg daily for 2 years. The primary outcome was time to onset of diabetic nephropathy, defined by persistent albuminuria in overnight specimens, with a urinary albumin excretion rate >200 mcg/min and at least 30% higher than the baseline level. Ten of the 194 patients in the 300-mg group (5.2%) and 19 of the 195 patients in the 150-mg group (9.7%) reached the primary endpoint, as compared with 30 of the 201 patients in the placebo group (14.9%) (HR 0.30, 95% CI 0.14–0.61, *p* < 0.001, and HR 0.61, 95% CI 0.34–1.08, *p* = 0.081 for the two irbesartan groups, respectively). The average blood pressure during the course of the study was 144/83 mmHg in the placebo group, 143/83 mmHg in the 150-mg group, and 141/83 mmHg in the 300-mg group (*p* = 0.004 for the comparison of systolic blood pressure between the placebo group and the combined irbesartan groups) [143].

*Treatment*

### 49. The choice of initial drug therapy for hypertension should be based on efficacy, tolerability, cost, and presence of comorbidities. In general, diuretics, ACE inhibitors, angiotensin receptor blockers, or calcium channel blockers can be useful as initial monotherapy. [IIa, B]

#### Summary of evidence

- The randomized, double-blind, active-controlled antihypertensive and lipid-lowering treatment to prevent heart attack trial (ALLHAT), conducted from February 1994 through March 2002, evaluated 33,357 participants (age ≥ 55 years) with hypertension and at least one additional CHD risk factor from 623 North American centers to determine if calcium channel blockers or angiotensin-converting enzyme (ACE) inhibitors would lower the incidence of CHD or other CV events vs. treatment with a diuretic. The primary outcome was combined fatal CHD or nonfatal myocardial infarction, analyzed by intention to treat. Secondary outcomes were all-cause mortality, stroke, combined CHD (primary outcome, coronary revascularization, or angina with hospitalization), and combined CVD (combined CHD, stroke, treated angina without hospitalization, heart failure, and peripheral arterial disease). The primary outcome occurred in 2956 participants, with no difference between treatments. Compared with chlortalidone (6-year rate, 11.5%), the relative risks (RRs) were 0.98 (95% CI 0.90–1.07) for amlodipine (6-year rate, 11.3%) and 0.99 (95% CI 0.91–1.08) for lisinopril (6-year rate, 11.4%). Likewise, all-cause mortality did not differ between groups. For amlodipine vs. chlortalidone, secondary outcomes were similar except for a higher 6-year rate of heart failure with amlodipine (10.2% vs. 7.7%; RR, 1.38; 95% CI 1.25–1.52). For lisinopril vs. chlortalidone, lisinopril had higher 6-year rates of combined CVD (33.3% vs. 30.9%; RR, 1.10; 95% CI 1.05–1.16); stroke (6.3% vs. 5.6%; RR, 1.15; 95% CI 1.02–1.30); and HF (8.7% vs. 7.7%; RR, 1.19; 95% CI 1.07–1.31) [144].
- An analysis of the ALLHAT study to determine if treatment with a calcium channel blocker or an ACE inhibitor would decrease clinical complications compared with treatment with a thiazide-type diuretic in DM, IFG, and normoglycemia provided no evidence of superiority for treatment with calcium channel blockers or ACE inhibitors compared with a thiazide-type diuretic during first-step antihypertensive therapy in these populations [145].
- A meta-analysis of 354 randomized, double-blind, placebo-controlled trials of thiazides, beta blockers, ACE inhibitors, angiotensin II receptor antagonists, and calcium channel blockers in fixed dose was performed. Placebo adjusted reductions in systolic and diastolic blood pressure and prevalence of adverse effects, according to dose expressed as a multiple of the standard (recommended) doses of the drugs, were the main outcomes. All five classes produced similar reductions in blood pressure, with average SBP and DBP reductions of 9.1 and 5.5 mmHg respectively at standard doses and 7.1 and 4.4 mmHg respectively (20% lower) at half-standard doses. The drugs reduced blood pressure from all pretreatment levels, more so from higher levels; for a 10 mmHg-higher blood pressure, the reduction was 1.0 mmHg greater in SBP and 1.1 mmHg greater in DBP. The BP-lowering effects of different drug classes were additive. In addition, combination low-dose treatment increased efficacy and reduced adverse effects. From the average blood pressure in people who have strokes (150/90 mmHg), three drugs at half-standard dose were estimated to lower blood pressure by 20 mmHg systolic and 11 mmHg diastolic, thereby reducing the risk of stroke by 63% and the risk of ischemic heart disease events by 46% in the 60–69 age range [146].

### 50. In patients with diabetes and urinary albumin >30 mg/g, treatment with ACE inhibitors or angiotensin receptor blockers are indicated. [I, A]

#### Summary of evidence

- The reduction in end points in noninsulin-dependent diabetes mellitus with the angiotensin II antagonist losartan (RENAAL) study [147] investigated if albuminuria, a marker of renal disease, could also be a monitor of the renoprotective efficacy of RAS intervention by the angiotensin II (Ang II) antagonist losartan in patients with diabetic nephropathy. Data from the a double-blind randomized RENAAL trial were used to examine the effects of losartan on a renal outcome (primary composite endpoint of doubling of serum creatinine, end-stage renal disease, or death) in 1513 type 2 diabetic patients with nephropathy. The effect of the degree of albuminuria at baseline, initial antiproteinuric response to therapy, and the degree of residual albuminuria on renal outcome (either the primary composite end point of RENAAL or ESRD) were examined, as well as the contribution to renal protection of the antiproteinuric effect of losartan independently of changes in blood pressure. Albuminuria was the predominant renal risk marker in patients with type 2 diabetic nephropathy on conventional treatment; the higher the albuminuria, the greater the renal risk. Reduction in albuminuria was associated with a proportional effect on renal protection; the greater the reduction, the greater the renal protection. Residual albuminuria while on therapy (month 6) was considered as a strong marker of renal outcome, as was baseline albuminuria. The antiproteinuric effect of losartan explained a major component of its specific renoprotective effect. Reduction of residual albuminuria to the lowest achievable level should be viewed as a goal for renoprotective treatments.
- The Irbesartan Diabetic Nephropathy Trial addressed whether associations between baseline proteinuria and proteinuria reduction by either irbesartan, amlodipine, or control for similar decrements in blood pressure would be related to the cumulative incidence of renal endpoints. The risk of kidney failure doubled for each doubling of baseline proteinuria level (HR 2.04, 95% CI 1.87–2.22, *p* < 0.001). For each halving of proteinuria level between baseline and 12 months on treatment, risk of kidney failure was reduced by more than half (HR 0.44, 95% CI 0.40–0.49, *p* < 0.001). The reduction in risk for kidney failure was significantly greater for irbesartan vs. amlodipine (*p* = 0.048), but not control (*p* = 0.245) for the same proportional change in proteinuria. Proteinuria reduction in the first 12 months of therapy with irbesartan was associated with 36% of the total renoprotective effect observed. Proteinuria reduction using an angiotensin receptor-blocking agent should be regarded as an important therapeutic goal for renoprotection [148].
- The MICRO-HOPE study investigated whether the ACE inhibitor ramipril could lower risk of cardiovascular and renal disease in patients with diabetes [149]. A subset of 3577 people with diabetes included in the Heart Outcomes Prevention Evaluation study, aged 55 years or older, with a previous cardiovascular event or at least one other cardiovascular risk factor, no clinical proteinuria, heart failure, or low ejection fraction, and who were not taking ACE inhibitors were randomly assigned to receive ramipril (10 mg/day) or placebo and vitamin E or placebo, according to a two-by-two factorial design. The combined primary outcome was myocardial infarction, stroke, or CV death. Overt nephropathy was a main outcome in a substudy. The study was interrupted 6 months earlier (after 4.5 years) because of a consistent benefit of ramipril compared with placebo. Ramipril lowered the risk of the combined primary outcome by 25% (95% CI 12–36, *p* = 0.0004), myocardial infarction by 22% (95% CI 6–36), stroke by 33% (95% CI 10–50), CV death by 37% (95% CI 21–51), total mortality by 24% (95% CI 8–37), revascularization by 17% (95% CI 2–30), and overt nephropathy by 24% (95% CI 3–40, *p* = 0.027). After adjustment for changes in SBP (2.4 mmHg) and DBP (1.0 mmHg), ramipril still lowered the risk of the combined primary outcome by 25% (95% CI 12–36, *p* = 0.0004). Ramipril was beneficial for cardiovascular events and overt nephropathy in people with diabetes. The study concluded that cardiovascular benefit was greater than that attributable to the decrease in blood pressure, thus representing a vasculoprotective and renoprotective effect for people with diabetes.

### 51. When using more than one antihypertensive to achieve target blood pressure, it is reasonable to combine either an ACE inhibitor or an ARB with a dihydropyridine calcium channel blocker. [IIa, B]

#### Summary of evidence

- The ACCOMPLISH (Avoiding Cardiovascular Events Through COMbination Therapy in Patients Living With Systolic Hypertension) substudy [150], was designed to determine which combination therapy in patients with hypertension and diabetes most effectively decreased cardiovascular events. The outcomes effects of the ACE inhibitor benazepril, combined with amlodipine (B+A) or hydrochlorothiazide (B+H), were analyzed separately in diabetic patients as a pre-specified endpoint. A total of 6946 patients with diabetes were randomized to treatment with B+A or B+H. A subgroup of 2842 diabetic patients at very high risk (previous CV events or stroke) was also analyzed, as were 4559 patients without diabetes. The primary endpoint was a composite of cardiovascular death, myocardial infarction, stroke, hospitalization for angina, resuscitated arrest, and coronary revascularization. In the full diabetes group, the mean achieved blood pressures in the B+A and B+H groups were 131.5/72.6 and 132.7/73.7 mmHg respectively; over 30 months of follow-up, there were 307 (8.8%) and 383 (11.0%) primary events (HR 0.79, 95% CI 0.68–0.92, *p* = 0.003). For the diabetic patients at very high risk, there were 195 (13.6%) and 244 (17.3%) primary events (HR 0.77, 95% CI 0.64–0.93, *p* = 0.007). In the non-diabetic patients, there were 245 (10.8%) and 296 (12.9%) primary events (HR 0.82, 95% CI 0.69–0.97, p = 0.020). In the diabetic patients, B+A therapy had clear coronary benefits on both acute clinical events (*p* = 0.013) and revascularizations (*p* = 0.024). In patients with diabetes and hypertension, the calcium channel blocker amlodipine is superior to the diuretic hydrochlorothiazide when added to a renin-angiotensin system blocker for reduction of cardiovascular events in patients with diabetes requiring management of hypertension.

### 52. A combination of 3 or more drugs (ACE inhibitor or ARB plus amlodipine and a thiazide diuretic) can be useful in achieving BP goals. [IIa, B]

#### Summary of evidence

- A meta-analysis by Psaty [151] summarized the available clinical trial evidence concerning the safety and efficacy of various antihypertensive therapies used as first-line agents in terms of major cardiovascular disease endpoints and all-cause mortality. Network meta-analysis was used to combine direct within-trial between-drug comparisons with indirect evidence from other trials. Indirect comparisons preserving within-trial randomized findings were constructed from trials that had one treatment in common. Data were combined from 42 clinical trials that included 192,478 patients randomized to 7 major treatment strategies, including placebo. For all outcomes, low-dose diuretics were superior to placebo for coronary heart disease (CHD; RR 0.79, 95% CI 0.69–0.92); congestive heart failure (CHF; RR 0.51, 95% CI 0.42–0.62); stroke (RR 0.71, 95% CI 0.63–0.81); CV events (RR 0.76, 95% CI 0.69–0.83); CV mortality (RR 0.81, 95% CI 0.73–0.92); and total mortality (RR 0.90, 95% CI 0.84–0.96). None of the first-line treatment strategies—beta blockers, ACE inhibitors, calcium channel blockers (CCBs), alpha blockers, and angiotensin receptor blockers—was significantly better than low-dose diuretics for any outcome. Compared with CCBs, low-dose diuretics were associated with reduced risks of CV events (RR 0.94, 95% CI 0.89–1.00) and CHF (RR 0.74, 95% CI 0.67–0.81). Compared with ACE inhibitors, low-dose diuretics were associated with reduced risks of CHF (RR 0.88, 95% CI 0.80–0.96), CV events (RR 0.94, 95% CI 0.89–1.00), and stroke (RR 0.86, 0.77–0.97). Compared with beta blockers, low-dose diuretics were associated with a reduced risk of CV events (RR 0.89, 95% CI 0.80–0.98). Compared with alpha blockers, low-dose diuretics were associated with reduced risks of CHF (RR 0.51, 95% CI 0.43–0.60) and CV events (RR 0.84, 95% CI 0.75–0.93). Blood pressure changes were similar between comparison treatments. Low-dose diuretics were the most effective first-line treatment for preventing the occurrence of CV-related morbidity and mortality.

### 53. A combination of an ACE inhibitor and an ARB or a renin blocker is NOT recommended, due to the greater risk of loss of renal function, syncope, and hyperkalemia. [III, A]

#### Summary of evidence

- The ALTITUDE study asked whether the use of aliskiren would reduce cardiovascular and renal events in patients with type 2 diabetes and chronic kidney disease, cardiovascular disease, or both. The trial was stopped prematurely after the second interim efficacy analysis, because, after a median follow-up of 32.9 months, the primary endpoint (composite of the time to CV death or a first occurrence of cardiac arrest with resuscitation; nonfatal myocardial infarction; nonfatal stroke; unplanned hospitalization for heart failure; end-stage renal disease, death attributable to kidney failure, or need for renal replacement therapy with no dialysis or transplantation available or initiated; or doubling of the serum creatinine level from baseline) had occurred in 783 patients (18.3%) assigned to aliskiren as compared with 732 (17.1%) assigned to placebo (HR 1.08, 95% CI 0.98–1.20, *p* = 0.12). Thus, data do not support the addition of aliskiren to standard therapy with renin-angiotensin system blockade in patients with type 2 diabetes who are at high risk of cardiovascular and renal events. In fact, aliskiren may even be harmful [152].
- The ASTRONAUT study [153] was designed to investigate whether adding aliskiren to standard therapy would reduce the rate of CV death or readmission among HHF (hospitalization for heart failure) patients. Eligible patients were aged ≥18 years, with left ventricular ejection fraction (LVEF) 40% or less, elevated natriuretic peptides (brain natriuretic peptide [BNP] ≥ 400 pg/mL or N-terminal pro-BNP [NT-proBNP] ≥ 1600 pg/mL), and signs and symptoms of fluid overload. All patients received 150 mg of aliskiren (increased to 300 mg as tolerated) or placebo daily, in addition to standard therapy. The study drug was continued after discharge for a median 11.3 months. The main outcome measures were CV death or HF rehospitalization at 6 months and 12 months. In total, 1639 patients were randomized, with 1615 patients included in the final efficacy analysis cohort (808 aliskiren, 807 placebo). At randomization, patients were receiving diuretics (95.9%), beta blockers (82.5%), ACE inhibitors or ARBs (84.2%), and mineralocorticoid receptor antagonists (57.0%). In total, 24.9% of patients receiving aliskiren (77 CV deaths, 153 HF readmissions) and 26.5% of patients receiving placebo (85 CV deaths, 166 HF readmissions) experienced the primary endpoint at 6 months (HR 0.92, 95% CI 0.76–1.12, *p* = 0.41). At 12 months, the event rates were 35.0% for the aliskiren group (126 CV deaths, 212 HF readmissions) and 37.3% for the placebo group (137 CV deaths, 224 HF readmissions; HR 0.93, 95% CI 0.79–1.09, *p* = 0.36). The rates of hyperkalemia, hypotension, and renal impairment/failure were higher in the aliskiren group than in the placebo arm. Among patients hospitalized for HF with reduced LVEF, initiation of aliskiren in addition to standard therapy did not reduce CV death or HF readmission at 6 months or 12 months after discharge.
- In patients who have vascular disease or high-risk diabetes without heart failure, ACE inhibitors reduce mortality and morbidity from cardiovascular causes, but the role of ARBs in such patients is unknown. The ACE inhibitor ramipril, the ARB telmisartan, and a combination of the two drugs in patients with vascular disease or high-risk diabetes were compared in the ONTARGET study [154]. A total of 8576 patients were assigned to receive 10 mg of ramipril per day, 8542 were assigned to receive 80 mg of telmisartan per day, and 8502 were assigned to receive both drugs. The primary composite outcome (death from cardiovascular causes, myocardial infarction, stroke, or hospitalization for heart failure) occurred in 16.5% of patients in the ramipril group, 16.7% in the telmisartan group, and 16.3% in the combined-therapy group (differences were not statistically significant). However, more adverse events were seen in patients randomized to combined therapy. In conclusion, the combination of the two drugs was associated with more adverse events, without increased benefit.

## Module 7: Rationale for antiplatelet therapy

### 54. In patients with diabetes without clinical atherosclerotic disease (CLAD), i.e., in primary prevention, antiplatelet therapy is generally not recommended. [III, A]

#### Summary of evidence

- Trials of antiplatelet therapy versus control included about 70,000 “high-risk” patients (with vascular disease or another condition implying an increased risk of occlusive vascular disease) and 30,000 “low-risk” subjects from the general population. Direct comparisons of different antiplatelet regimens involved about 10,000 high-risk patients. In each of four main high-risk categories, antiplatelet therapy was definitely protective. Among low-risk recipients of “primary prevention”, a significant, one-third reduction in nonfatal myocardial infarction was accompanied by a nonsignificant increase in stroke. The absolute reduction in vascular events was much smaller than for high-risk patients, despite a much longer treatment period, and was not significant. There is no clear evidence on the balance of antiplatelet therapy in primary prevention among low-risk subjects [155].
- A meta-analysis of randomized controlled trials was performed to evaluate the benefits and harms of low-dose aspirin in people with diabetes and no cardiovascular disease. Six studies were eligible, with 10,117 participants. When aspirin was compared with placebo, there was no statistically significant reduction in the risk of major CV events (five studies, n = 9584; RR 0.90, 95% CI 0.81–1.00), CV mortality (four studies, n = 8557; RR 0.94, 95% CI 0.72–1.23), or all-cause mortality (four studies, n = 8557; RR 0.93, 95% CI 0.82–1.05). There was significant heterogeneity in analysis for myocardial infarction (I^2^ = 62.2%; *p* = 0.02) and stroke (I^2^ = 52.5%; *p* = 0.08). Aspirin significantly reduced the risk of myocardial infarction in men (0.57, 0.34–0.94) but not in women (1.08, 0.71–1.65; *p* value for interaction = 0.056). Evidence relating to harms was inconsistent [156].

### 55. In patients with diabetes without clinical atherosclerotic disease (Table 5) in the HIGH-RISK category (Table 2), aged >65 years and with low risk of bleeding, acetylsalicylic acid can be useful. [IIa, B]

#### Summary of evidence

- The Japanese Primary Prevention of Atherosclerosis with Aspirin for Diabetes (JPAD) trial was designed to examine the efficacy of low-dose aspirin for primary prevention of atherosclerotic events in patients with type 2 diabetes and no previous cardiovascular events. The study randomized 1262 patients to receive aspirin (81 mg or 100 mg) and 1277 patients to a non-aspirin group. Mean (SD) age was 65 (10) years, and 55% were men; 58% of patients had hypertension, 53% had dyslipidemia, and BP and HbA1c were well controlled in both groups. The median follow-up period was 4.37 years and 193 patients were lost to follow-up, with data for those patients censored at the day of last follow-up. There was no reduction in the risk of CV events with low-dose aspirin for high-risk patients with diabetes in primary prevention. However, the event rate was lower than expected overall, and these findings should be interpreted in context with the low incidence of atherosclerotic disease in Japan and current management of cardiovascular risk factors [157].
- A sub-analysis from the JPAD study evaluating patients with diabetes divided according to SBP and DBP at enrollment (“target unattained” group, SBP ≥ 140 mmHg and/or DBP ≥ 90 mmHg; “target attained” group, SBP < 140 mmHg and DBP < 90 mmHg) demonstrated that the incidence of the primary atherosclerotic events, especially cerebrovascular events, was higher in the unattained group than in the attained group. The incidence of cerebrovascular events was higher in the unattained group than in the attained group in patients without aspirin therapy; however, the incidence of cerebrovascular events in the unattained group was as low as the incidence in the attained group in patients on aspirin therapy. Cox proportional hazards analysis revealed that BP level was an independent predictor of cerebrovascular events in diabetic patients [158].
- In a meta-analysis of RCTs with aspirin including 14 trials (107,686 participants), aspirin was associated with reductions in major cardiovascular events (risk ratio 0.90, 95% CI 0.85–0.95), myocardial infarction (risk ratio 0.86, 95% CI 0.75–0.93), ischemic stroke (risk ratio 0.86, 95% CI 0.75–0.98), and all-cause mortality (risk ratio 0.94, 95% CI 0.89–0.99). However, there were increases in hemorrhagic stroke (risk ratio 1.34, 95% CI 1.01–1.79) and major bleeding (risk ratio 1.55, 95% CI 1.35–1.78) with aspirin. The number needed to treat to prevent 1 major cardiovascular event over a mean follow-up of 6.8 years was 284. By comparison, the number needed to harm to cause 1 major bleeding was 299. In subgroup analyses, pooled results demonstrated a reduction in myocardial infarction among men (RR 0.71, 95% CI 0.59–0.85) and ischemic stroke among women (RR 0.77, 95% CI 0.63–0.93). Aspirin use was associated with a reduction (RR 0.65, 95% CI 0.51–0.82) in myocardial infarction among diabetic men. The results of meta-regression analyses suggested that aspirin therapy might be associated with a decrease in stroke among diabetic women and a decrease in MI among diabetic men, and that risk reductions achieved with low doses (75 mg/day) were as large as those obtained with higher doses (650 mg/day). The study concluded that low-dose aspirin was beneficial for primary prevention of CVD, and that the decision regarding an aspirin regimen should be made on an individual patient basis. The effects of aspirin therapy varied by sex and diabetes status [159].

### 56. In VERY HIGH-RISK patients, including those with clinical atherosclerotic disease (CLAD) and prior cardiovascular events (secondary prevention), antiplatelet therapy is indicated. [I, A]

#### Summary of evidence

- A collaborative meta-analysis of randomized trials of an antiplatelet regimen versus control or of one antiplatelet regimen versus another in high-risk patients (with acute or previous vascular disease or some other predisposing condition) was performed [6]. Trials had to use a randomization method that precluded prior knowledge of the next treatment to be allocated, and comparisons had to have study groups that differed only in terms of antiplatelet regimen. A total of 287 studies were included, involving 135,000 patients in comparisons of antiplatelet therapy versus control and 77,000 in comparisons of different antiplatelet regimens. Aspirin (or another oral antiplatelet drug) was protective in most types of patient at increased risk of occlusive vascular events, including those with AMI or ischemic stroke, unstable or stable angina, previous MI, stroke or cerebral ischemia, peripheral arterial disease, or atrial fibrillation [160].

### 57. In VERY HIGH-RISK patients with aspirin allergy or gastric intolerance, clopidogrel should be considered as an acceptable alternative. [IIa, B]

#### Summary of evidence

- The Clopidogrel vs. Aspirin in Patients at Risk of Ischemic Events (CAPRIE) trial was a randomized, blinded, multicenter trial of 19,185 patients with atherosclerotic disease manifested as recent ischemic stroke or myocardial infarction or symptomatic peripheral arterial disease. The number of readmissions for ischemic events (defined as angina, transient ischemic attack, or limb ischemia) or bleeding events was determined for the entire cohort. A significant reduction in the total number of readmissions for ischemic events or bleeding was seen with clopidogrel use compared with aspirin (1502 vs. 1673, *p* = 0.010) over an average of 1.6 years of treatment. This reduction in rehospitalization was consistent across individual outcomes of angina, transient ischemic attack, limb ischemia, and bleeding. Clopidogrel also resulted in a 7.9% relative risk reduction in a combined endpoint of vascular death, stroke, myocardial infarction, or rehospitalization for ischemic events or bleeding (15.1–13.7% at 1 year, *p* = 0.011) as compared with aspirin [161].

### 58. Dual antiplatelet therapy is recommended for at least 1 year in VERY HIGH-RISK patients after acute coronary syndrome. [I, A]

#### Summary of evidence

- The TRITON-TIMI 38 trial aimed to randomly compare prasugrel (a new thienopyridine antiplatelet agent) vs. clopidogrel in 13,608 patients with moderate-to-high-risk acute coronary syndromes scheduled to undergo percutaneous coronary intervention. Prasugrel was given in a 60-mg loading dose and a 10-mg daily maintenance dose, while clopidogrel was given as a 300-mg loading dose and a 75-mg daily maintenance dose, for 6–15 months. The primary efficacy endpoint was death from cardiovascular causes, nonfatal myocardial infarction, or nonfatal stroke. The key safety endpoint was major bleeding. The primary endpoint occurred in 12.1% of patients in the clopidogrel arm and 9.9% of patients receiving prasugrel (HR for prasugrel vs. clopidogrel, 0.81; 95% CI 0.73–0.90, *p* < 0.001). In the prasugrel group, there were significant reductions in rates of myocardial infarction (9.7% for clopidogrel vs. 7.4% for prasugrel, *p* < 0.001), urgent target-vessel revascularization (3.7% vs. 2.5%, *p* < 0.001), and stent thrombosis (2.4% vs. 1.1%, *p* < 0.001). Major bleeding was observed in 2.4% of patients receiving prasugrel and in 1.8% of patients receiving clopidogrel (HR 1.32, 95% CI 1.03–1.68, *p* = 0.03). The rate of life-threatening bleeding was higher in the prasugrel arm (1.4% vs. 0.9%, *p* = 0.01), including nonfatal (1.1% vs. 0.9%, HR 1.25, *p* = 0.23) and fatal bleeding (0.4% vs. 0.1%, *p* = 0.002) [162].
- In the CURE study, 2658 patients with non-ST-elevation acute coronary syndrome undergoing PCI were randomly assigned to double-blind treatment with clopidogrel (*n* = 1313) or placebo (*n* = 1345). Patients were pretreated with aspirin and clopidogrel for a median of 6 days before PCI during the initial hospital admission, and for a median of 10 days overall. After PCI, most patients (>80%) in both groups received open-label thienopyridine for about 4 weeks, after which clopidogrel was restarted for a mean of 8 months. The primary endpoint was a composite of cardiovascular death, myocardial infarction, or urgent target-vessel revascularization within 30 days of PCI, in an intention-to-treat analysis. Fifty-nine (4.5%) patients in the clopidogrel group reached the primary endpoint, compared with 86 (6.4%) in the placebo group (RR 0.70, 95% CI 0.50–0.97, *p* = 0.03). Long-term administration of clopidogrel after PCI was associated with a lower rate of CV death, myocardial infarction, or any revascularization (*p* = 0.03) and of CV death or myocardial infarction (*p* = 0.047). Overall, including events before and after PCI, there was a 31% reduction in CV death or myocardial infarction (*p* = 0.002), and, at follow-up, there was no significant difference in major bleeding between the groups (*p* = 0.64) [163].
- In the CHARISMA trial, 15,603 patients with either clinically evident cardiovascular disease or multiple risk factors were randomized to receive clopidogrel (75 mg per day) plus low-dose aspirin (75–162 mg per day) or placebo plus low-dose aspirin and followed for a median of 28 months. The primary endpoint was a composite of myocardial infarction, stroke, or death from cardiovascular causes. The rate of the primary efficacy endpoint was 6.8% with clopidogrel plus aspirin and 7.3% with placebo plus aspirin (RR 0.93, 95% CI 0.83–1.05, *p* = 0.22). The principal secondary efficacy endpoint, which included hospitalizations for ischemic events, occurred in 16.7% and 17.9% (RR 0.92, 95% CI 0.86–0.995, *p* = 0.04), and the rate of severe bleeding was 1.7% and 1.3% (RR 1.25, 95% CI 0.97–1.61. *p* = 0.09). In patients with multiple risk factors, the rate of the primary endpoint was 6.6% with clopidogrel and 5.5% with placebo (RR 1.2, 95% CI 0.91–1.59, *p* = 0.20), and the rate of death from cardiovascular causes was also higher with clopidogrel (3.9% vs. 2.2%, p = 0.01). In subgroup analysis of patients with clinically evident atherothrombosis, the rate was 6.9% with clopidogrel and 7.9% with placebo (RR 0.88, 95% CI 0.77–0.998, *p* = 0.046), suggesting benefit with clopidogrel treatment in patients with symptomatic atherothrombosis and harm in patients with multiple risk factors. Overall, clopidogrel plus aspirin was not significantly more effective than aspirin alone in reducing the rate of myocardial infarction, stroke, or death from cardiovascular causes [164].
- In the multicenter, double-blind, randomized PLATO trial, ticagrelor (180-mg loading dose, 90 mg twice daily thereafter) and clopidogrel (300–600-mg loading dose, 75 mg daily thereafter) were compared for the prevention of CV events in 18,624 patients admitted to hospital with an acute coronary syndrome, with or without ST-segment elevation. At 12 months, the primary composite endpoint (death from vascular causes, myocardial infarction, or stroke) had occurred in 9.8% of patients receiving ticagrelor as compared with 11.7% of those receiving clopidogrel (*p* < 0.001). The rate of death from any cause was also reduced with ticagrelor (4.5% vs. 5.9% with clopidogrel, *p* < 0.001). No significant difference in rates of major bleeding was found between the ticagrelor and clopidogrel groups (*p* = 0.43), but ticagrelor was associated with a higher rate of major bleeding not related to coronary-artery bypass grafting (4.5% vs. 3.8%, *p* = 0.03), including more instances of fatal intracranial bleeding and fewer of fatal bleeding of other types [165].
- The PEGASUS study investigated the efficacy and safety of ticagrelor after an acute coronary syndrome, in a double-blind 1:1:1 fashion. The trial randomized 21,162 patients who had had a myocardial infarction 1–3 years earlier to ticagrelor 90 mg twice daily, ticagrelor 60 mg twice daily, or placebo. All patients received low-dose aspirin and were followed for a median of 33 months. The primary efficacy endpoint was the composite of cardiovascular death, myocardial infarction, or stroke. The primary safety endpoint was Thrombolysis in Myocardial Infarction (TIMI)-defined major bleeding. Both ticagrelor doses reduced the rate of the primary efficacy endpoint, with Kaplan–Meier rates at 3 years of 7.85% in the 90-mg ticagrelor group, 7.77% in the 60-mg ticagrelor group, and 9.04% in the placebo group (HR for 90-mg ticagrelor vs. placebo: 0.85, 95% CI 0.75–0.96, *p* = 0.008; HR for 60-mg ticagrelor vs. placebo: 0.84, 95% CI 0.74–0.95, *p* = 0.004). Rates of TIMI major bleeding were higher with ticagrelor (2.60% with 90 mg and 2.30% with 60 mg) than with placebo (1.06%) (*p* < 0.001 for each dose vs. placebo); the rates of intracranial hemorrhage or fatal bleeding in the three groups were 0.63, 0.71, and 0.60% respectively. Therefore, in patients with a previous myocardial infarction at least 1 year before, treatment with ticagrelor significantly reduced the risk of CV death, myocardial infarction, and stroke, but increased the risk of major bleeding [166].

### 59. In patients who are not at high risk of bleeding complications, continuation of dual antiplatelet therapy may be reasonable for longer than 12 months after acute coronary syndrome. [IIb, A]

#### Summary of evidence

- In the PEGASUS study, the efficacy and safety of ticagrelor after an acute coronary syndrome was investigated in a double-blind 1:1:1 fashion. The trial randomized 21,162 patients who had had a myocardial infarction 1–3 years earlier to ticagrelor at a dose of 90 mg twice daily, ticagrelor at a dose of 60 mg twice daily, or placebo. All the patients received low-dose aspirin and were followed for a median of 33 months. The primary efficacy endpoint was the composite of cardiovascular death, myocardial infarction, or stroke. The primary safety endpoint was Thrombolysis in Myocardial Infarction (TIMI) major bleeding. Ticagrelor in both doses reduced the rate of the primary efficacy endpoint, with Kaplan–Meier rates at 3 years of 7.85% in the group receiving 90 mg of ticagrelor twice daily, 7.77% in the group receiving 60 mg of ticagrelor twice daily, and 9.04% in the placebo group (hazard ratio for 90 mg of ticagrelor vs. placebo, 0.85; 95% CI 0.75–0.96; *p* = 0.008; hazard ratio for 60 mg of ticagrelor vs. placebo, 0.84; 95% CI 0.74–0.95; *p* = 0.004). Rates of TIMI major bleeding were higher with ticagrelor (2.60% with 90 mg and 2.30% with 60 mg) than with placebo (1.06%) *(p* < 0.001 for each dose vs. placebo); the rates of intracranial hemorrhage or fatal bleeding in the three groups were 0.63, 0.71, and 0.60% respectively. Therefore, in patients with a previous myocardial infarction, at least one year earlier, treatment with ticagrelor significantly reduced the risk of cardiovascular death, myocardial infarction, or stroke, and increased the risk of major bleeding [166]. The DAPT study sought to investigate if 30 months of DAPT was superior to 12 months in patients undergoing DES and bare-metal stent (BMS) PCI. A total of 9961 patients were randomized at 452 sites in 11 countries: 5020 to prolonged DAPT and 4941 to placebo. Approximately 30% had diabetes mellitus, 25% were smokers and 6% had peripheral arterial disease. Patients were enrolled 72 h after stent placement and were given open-label aspirin and thienopyridine for 12 months, per current practice norms. Indication for PCI was stable angina in 38%, ST-segment elevation myocardial infarction (STEMI) in 10% and NSTE-acute coronary syndrome (NSTE-ACS) in 32%. Approximately two-thirds of the patients received clopidogrel, whereas the rest received prasugrel. At 12 months, patients without an ischemic or bleeding complication and with documented compliance, were randomized in a 1:1 fashion to receive an additional 18 months of DAPT or matching placebo. The primary endpoint of major adverse cardiac and cerebrovascular events (MACCE) was significantly lower in the continued DAPT arm compared with placebo (4.3% vs. 5.9%, hazard ratio 0.71, 95% confidence interval 0.59–0.85, *p* < 0.001). There were reductions in all MI (2.1% vs. 4.1%, *p* < 0.001) and stent thrombosis (0.4% vs. 1.4%, *p* < 0.001), but all-cause mortality was higher (2.0% vs. 1.5%, *p* = 0.05), driven mostly by an increase in non-cardiovascular deaths (1% vs. 0.5%, *p* = 0.002), including cancer-related death (0.62% vs. 0.28%, *p* = 0.02) and bleeding-related death (0.22% vs. 0.06%, *p* = 0.06). Moderate and severe GUSTO bleeding was also higher with DAPT (2.5% vs. 1.6%, *p* = 0.001), as was BARC 2, 3, or, 5 bleeding (5.6% vs. 2.9%, *p* < 0.001). The DAPT study showed that longer duration of DAPT following PCI results in lower stent thrombosis and recurrent MIs, but higher bleeding and all-cause mortality compared with a 12-month duration [167].

## Conclusions

Although cardiovascular risk is increased in patients with diabetes when compared to age-matched nondiabetic individuals, recent evidence indicates that there is a high prevalence of lower-risk individuals among this population. Risk stratification is clearly needed, either to intensify more effective preventive measures in high-risk categories or to avoid overtreatment of lower-risk patients. The present Panel structured a risk-based guide to help clinicians optimize cardiovascular prevention in diabetes. The Panel recovered the concept of treating-to-target, as they are considered important to promote better adhesion to treatment and can be useful for clinicians to improve prevention in clinical practice. In the present guideline, there is a clear shift toward a more intensive treatment in the very-high risk category, especially regarding lipid-lowering therapy with statins, where new, lower lipid targets are proposed. The Panel understands that patients with diabetes at very high risk have very high mortality and one of the most important currently available actions to reduce residual risk is to obtain further reductions in LDL-c levels. The panel also reviews the potential role of the new anti-hyperglycemic drugs in reducing cardiovascular risk, as well as hypertension targets and drug choice. Finally, we also propose a practical guideline to guide decision-making about screening for silent coronary artery disease. We understand that intensifying treatment may increase costs to the health care system; however, the number of avoided events and lives saved clearly outweighs these costs. The Brazilian Diabetes Society, the Brazilian Cardiology Society, and the Brazilian Endocrinology Society are now united in the task to reduce cardiovascular disease in patients with diabetes.

## Abbreviations

4D: die deutsche diabetes dialyze
ABI: ankle-brachial index
ACCOMPLISH: Avoiding Cardiovascular Events Through COMbination Therapy in Patients Living With Systolic Hypertension
ACCORD: action to control cardiovascular risk in diabetes
ACCORD-LIPID: action to control cardiovascular risk in diabetes-lipids arm
ACE: angiotensin-converting enzyme
ADVANCE: action in diabetes and vascular disease: preterax and diamicron modified release controlled evaluation
AMI: acute myocardial infarction
ARIC: atherosclerosis risk in communities
AUC-ROC: area under the ROC curve
AURORA: a study to evaluate the use of rosuvastatin in subjects on regular hemodialysis
BMS: bare-metal stent
CAC: coronary artery calcium
CAD: coronary artery disease
CAPRIE: clopidogrel vs. aspirin in patients at risk of ischemic events
CARDIA: coronary artery risk development in young adults
CARDS: Collaborative Atorvastatin Diabetes Study
CCBs: calcium channel blockers
CCTA: coronary computed tomography angiography
CHD: coronary heart disease
CHF: congestive heart failure
CIMT: carotid-artery intima-media thickness
CKD: chronic kidney disease
CLAD: clinical atherosclerotic disease
CMRI: cardiac magnetic resonance imaging
CORONA: controlled rosuvastatin multinational trial in heart failure
cPB: carotid plaque burden
CV: cardiovascular
CVD: cardiovascular disease
DCCT: diabetes control and complications trial
DIAD: detection of ischemia in asymptomatic diabetics
DYNAMIT: do you need to assess myocardial ischemia in type-2 diabetes
ECG: electrocardiogram
EDIC: epidemiology of diabetes interventions and complications
ESRD: end-stage renal disease
FH: familiar hypercholesterolemia
FIELD: fenofibrate intervention and event lowering in diabetes
GISSI-HF: effect of rosuvastatin in patients with chronic heart failure
HHF: hospitalization for heart failure
HR: hazard ratios
ICU: intensive care unit
INVEST: International Verapamil-Trandolapril Study
ISH: isolated systolic hypertension
JPAD: Japanese Primary Prevention of Atherosclerosis with Aspirin for Diabetes
LVEF: left ventricular ejection fraction
MACCE: major adverse cardiac and cerebrovascular events
MACE: major adverse cardiovascular events
MI: myocardial infarction
MPI: myocardial perfusion imaging
MPS: myocardial perfusion SPECT
NRI: net reclassification index
NSTE-ACS: NSTE-acute coronary syndrome
PCSK9: proprotein convertase subtilisin–kexin type 9 serine protease
RAS: renin-angiotensin system
RCTs: randomized controlled trials
RR: rate ratio
RRR: relative risk reduction
SCAT: subclinical atherosclerosis
SMI: silent myocardial ischemia
SPECT: single-photon emission computed tomography
STEMI: ST-segment elevation myocardial infarction
TIMI: thrombolysis in myocardial infarction
TNT: treat to new targets
UKPDS: United Kingdom Prospective Diabetes Study
VADT: veterans affairs diabetes trial
